# Supplementary figures and images for: Development and validation of the Multidimensional Internally Regulated Eating Scale (MIRES)
Source: PLoS One. 2020 Oct 8;15(10):e0239904. doi: 10.1371/journal.pone.0239904 (PMC7544044; doi:10.1371/journal.pone.0239904)

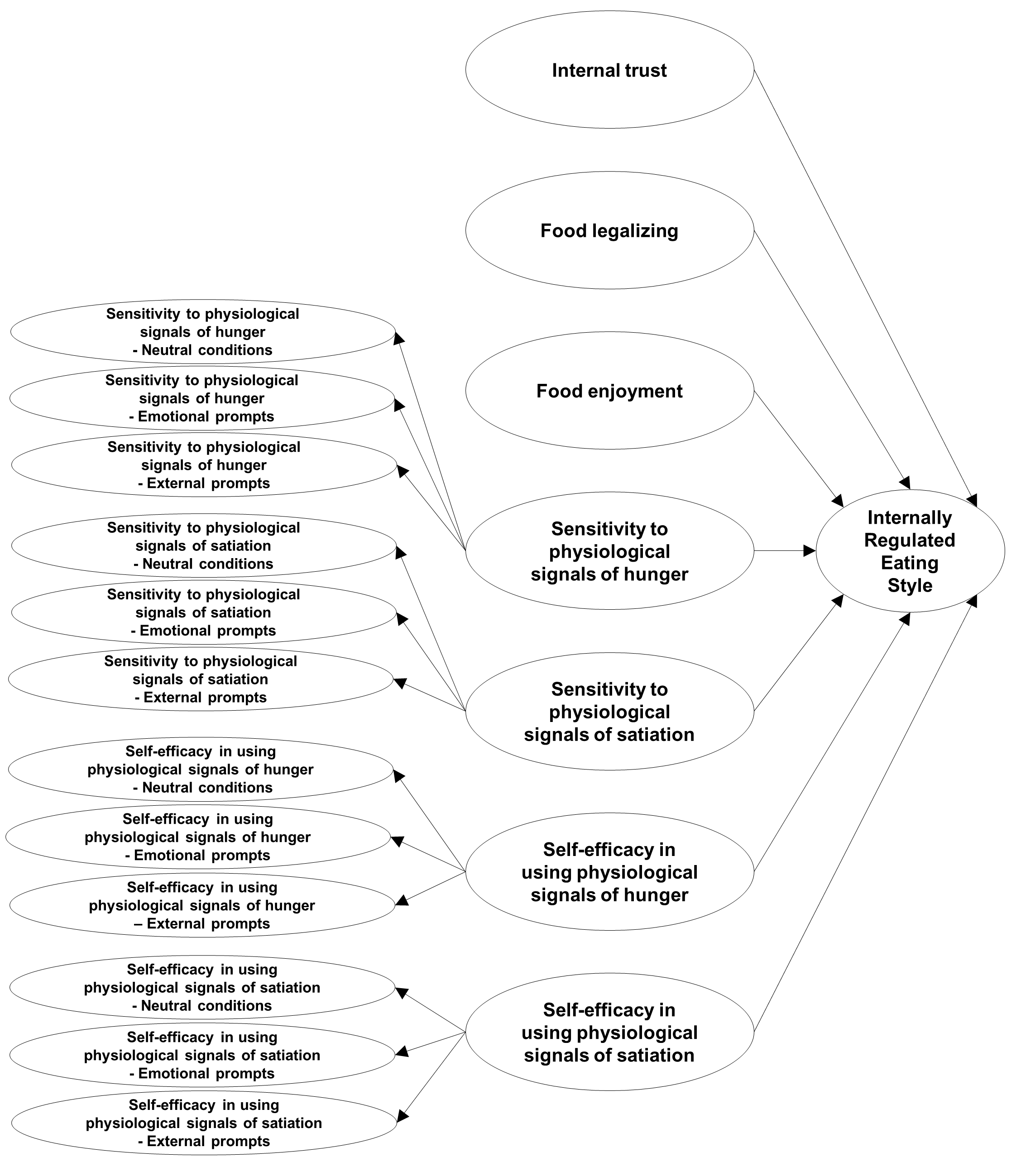

Supplement: S1 Fig — The direction of arrows indicates whether a construct is formative—arrows point to the construct—or reflective—arrows point to the dimension. (TIF) [file pone.0239904.s001.tif]

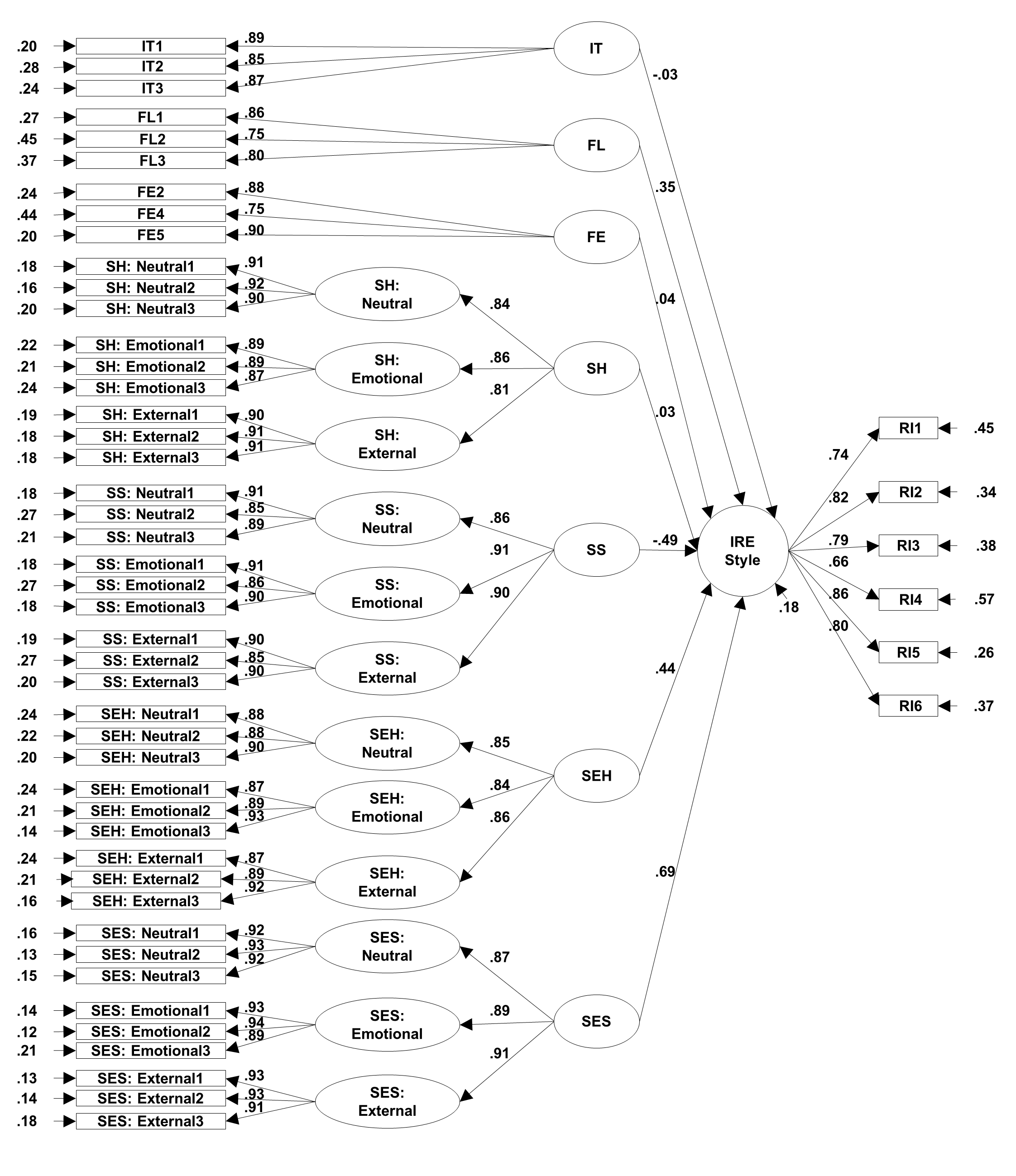

Supplement: S2 Fig — All loadings were significant at the 0.01 level. Context effects, method effects, covariances between first- and second-order factors, and disturbance terms of first- and second-order factors are not depicted in the figure for easier readability. (TIF) [file pone.0239904.s002.tif]
