# Supplementary material for: Development and validation of the Multidimensional Internally Regulated Eating Scale (MIRES)
Source: PLoS One. 2020 Oct 8;15(10):e0239904. doi: 10.1371/journal.pone.0239904 (PMC7544044; doi:10.1371/journal.pone.0239904)
Supplement: S1 Table — /SI_Caption> (DOCX) [file pone.0239904.s003.docx]

# **S1 Table.** **Factor loadings for the MIRES first- and second-order factors**

|  | Item/Factor | Standardized loading |
| --- | --- | --- |
| First-order factors | | |
| IT | IT1  IT2  IT3  IT4 | 0.92  0.87  0.89  0.90 |
| FL | FL1  FL2  FL3  FL4 | 0.83  0.73  0.90  0.90 |
| FE | FE1  FE2  FE3  FE4  FE5 | 0.92  0.86  0.83  0.80  0.91 |
| SH: Neutral | SH: Neutral1  SH: Neutral2  SH: Neutral3 | 0.83  0.88  0.81 |
| SH: Emotional | SH: Emotional1  SH: Emotional2  SH: Emotional3 | 0.85  0.83  0.82 |
| SH: External | SH: External1  SH: External2  SH: External3 | 0.83  0.88  0.84 |
| SS: Neutral | SS: Neutral1  SS: Neutral2  SS: Neutral3 | 0.89  0.79  0.87 |
| SS: Emotional | SS: Emotional1  SS: Emotional2  SS: Emotional3 | 0.90  0.83  0.90 |
| SS: External | SS: External1  SS: External2  SS: External3 | 0.88  0.79  0.87 |
| SEH: Neutral | SEH: Neutral1  SEH: Neutral2  SEH: Neutral3 | 0.85  0.86  0.89 |
| SEH: Emotional | SEH: Emotional1  SEH: Emotiona2l  SEH: Emotional3 | 0.85  0.84  0.90 |
| SEH: External | SEH: External1  SEH: External2  SEH: External3 | 0.85  0.87  0.90 |
| SES: Neutral | SES: Neutral1  SES: Neutral2  SES: Neutral3 | 0.93  0.94  0.89 |
| SES: Emotional | SES: Emotional1  SES: Emotional2  SES: Emotional3 | 0.94  0.95  0.92 |
| SES: External | SES: External1  SES: External2  SES: External3 | 0.92  0.92  0.89 |
| Second-order factors | | |
| SH | SH: Neutral  SH: Emotional  SH: External | 0.80  0.81  0.81 |
| SS | SS: Neutral  SS: Emotional  SS: External | 0.85  0.89  0.94 |
| SEH | SEH: Neutral  SEH: Emotional  SEH: External | 0.84  0.84  0.87 |
| SES | SES: Neutral  SES: Emotional  SES: External | 0.87  0.90  0.95 |

IT: Internal trust, FL: Food legalizing, FE: Food enjoyment, SH: Sensitivity to physiological signals of hunger, SS: Sensitivity to physiological signals of satiation, SEH: Self-efficacy in using physiological signals of hunger, SES: Self-efficacy in using physiological signals of satiation.
