# Supplementary material for: Development and validation of the Multidimensional Internally Regulated Eating Scale (MIRES)
Source: PLoS One. 2020 Oct 8;15(10):e0239904. doi: 10.1371/journal.pone.0239904 (PMC7544044; doi:10.1371/journal.pone.0239904)
Supplement: S2 Table — (DOCX) [file pone.0239904.s004.docx]

# **S2 Table. Mean scores on MIRES first- and second-order factors for dieters and non-dieters.**

|  | Mean  Dieters  (n_1_ = 131) | Mean  Non-dieters  (n_2_ = 843) | t value | P value | Cohen's d |  |
| --- | --- | --- | --- | --- | --- | --- |
| First-order factors | | | | | | |
| IT | 3.38 | 4.69 | -8.63 | < 0.001 | -0.81 |  |
| FL | 3.05 | 4.64 | -9.92 | < 0.001 | -0.93 |  |
| FE | 5.24 | 5.35 | -.89 | 0.376 | -0.08 |  |
| SH: Neutral | 5.58 | 5.96 | -3.76 | < 0.001 | -0.35 |  |
| SH: Emotional | 5.04 | 5.44 | -2.90 | 0.004 | -0.27 |  |
| SH: External | 4.81 | 5.40 | -4.46 | < 0.001 | -0.42 |  |
| SS: Neutral | 4.85 | 5.66 | -5.85 | < 0.001 | -0.55 |  |
| SS: Emotional | 3.89 | 4.97 | -6.84 | < 0.001 | -0.64 |  |
| SS: External | 4.27 | 5.21 | -6.67 | < 0.001 | -0.63 |  |
| SEH: Neutral | 4.95 | 5.57 | -5.05 | < 0.001 | -0.47 |  |
| SEH: Emotional | 4.29 | 4.94 | -4.30 | < 0.001 | -0.40 |  |
| SEH: External | 4.33 | 5.11 | -5.56 | < 0.001 | -0.52 |  |
| SES: Neutral | 4.45 | 5.48 | -6.37 | < 0.001 | -0.60 |  |
| SES: Emotional | 3.59 | 4.86 | -7.49 | < 0.001 | -0.70 |  |
| SES: External | 4.06 | 5.18 | -7.40 | < 0.001 | -0.70 |  |
| Second-order factors | | | | | | |
| SH | 5.14 | 5.60 | -4.73 | < 0.001 | -0.44 |  |
| SS | 4.34 | 5.28 | -7.41 | < 0.001 | -0.70 |  |
| SEH | 4.52 | 5.21 | -5.64 | < 0.001 | -0.53 |  |
| SES | 4.03 | 5.17 | -8.01 | < 0.001 | -0.75 |  |

IT: Internal trust, FL: Food legalizing, FE: Food enjoyment, SH: Sensitivity to physiological signals of hunger, SS: Sensitivity to physiological signals of satiation, SEH: Self-efficacy in using physiological signals of hunger, SES: Self-efficacy in using physiological signals of satiation.
