# Supplementary material for: Development and validation of the Multidimensional Internally Regulated Eating Scale (MIRES)
Source: PLoS One. 2020 Oct 8;15(10):e0239904. doi: 10.1371/journal.pone.0239904 (PMC7544044; doi:10.1371/journal.pone.0239904)
Supplement: S3 Table — (DOCX) [file pone.0239904.s005.docx]

# **S3 Table. Additional sample characteristics of the US sample.**

|  | Male  *(N = 590)* | Female  *(N = 610)* | Total  *(N = 1200*) |
| --- | --- | --- | --- |
| Household composition |  |  |  |
| With children | 219 (18.3) | 287 (23.9) | 506 (42.2) |
| Without children | 371 (30.9) | 323 (26.9) | 694 (57.8) |
| BMI group (N = 1198) |  |  |  |
| Underweight (<18.5) | 29 (2.4) | 30 (2.5) | 59 (4.9) |
| Normal (18.5-24.9) | 170 (14.2) | 198 (16.5) | 368 (30.7) |
| Overweight (25.0-29.9) | 200 (16.7) | 155 (12.9) | 355 (29.6) |
| Obese (>30.0) | 189 (15.8) | 227 (19.0) | 416 (34.7) |
| Weight trajectory (N = 1189) |  |  |  |
| Stable weight | 43 (3.6) | 60 (5.0) | 103 (8.7) |
| Weight gain | 170 (14.3) | 151 (12.7) | 321 (27.0) |
| Weight loss | 132 (11.1) | 129 (10.9) | 261 (22.0) |
| Weight cycling | 240 (20.2) | 264 (22.2) | 504 (42.4) |
| History of eating disorders |  |  |  |
| Yes | 49 (4.1) | 39 (3.3) | 88 (7.3) |
| No | 541 (45.1) | 571 (47.6) | 1112 (92.7) |
| Currently following eating rules |  |  |  |
| Yes | 216 (18.0) | 232 (19.3) | 448 (37.3) |
| No | 374 (31.2) | 378 (31.5) | 752 (62.7) |

Values are presented as counts (percentages).
