# Supplementary material for: Development and validation of the Multidimensional Internally Regulated Eating Scale (MIRES)
Source: PLoS One. 2020 Oct 8;15(10):e0239904. doi: 10.1371/journal.pone.0239904 (PMC7544044; doi:10.1371/journal.pone.0239904)
Supplement: S4 Table — (DOCX) [file pone.0239904.s006.docx]

# **S4 Table. Bivariate correlations of summed scores of MIRES, RI, and MIRES subscales**

|  | 1 | 2 | 3 | 4 | 5 | 6 | 7 | 8 | 9 |
| --- | --- | --- | --- | --- | --- | --- | --- | --- | --- |
| 1. MIRES | - |  |  |  |  |  |  |  |  |
| 2. RI | 0.80^*^ | - |  |  |  |  |  |  |  |
| 3. IT | 0.71^*^ | 0.60^*^ | - |  |  |  |  |  |  |
| 4. FL | 0.63^*^ | 0.64^*^ | 0.61^*^ | - |  |  |  |  |  |
| 5. FE | 0.60^*^ | 0.46^*^ | 0.49^*^ | 0.38^*^ | - |  |  |  |  |
| 6. SH | 0.89^*^ | 0.66^*^ | 0.53^*^ | 0.43^*^ | 0.52^*^ | - |  |  |  |
| 7. SS | 0.95^*^ | 0.73^*^ | 0.61^*^ | 0.55^*^ | 0.49^*^ | 0.81^*^ | - |  |  |
| 8. SEH | 0.92^*^ | 0.74^*^ | 0.59^*^ | 0.49^*^ | 0.50^*^ | 0.85^*^ | 0.82^*^ | - |  |
| 9. SES | 0.93^*^ | 0.75^*^ | 0.60^*^ | 0.55^*^ | 0.45^*^ | 0.74^*^ | 0.91^*^ | 0.81^*^ | - |

MIRES: Multidimensional Internally Regulated Eating Scale, RI: Reflective items, IT: Internal trust, FL: Food legalizing, FE: Food enjoyment, SH: Sensitivity to physiological signals of hunger, SS: Sensitivity to physiological signals of satiation, SEH: Self-efficacy in using physiological signals of hunger, SES: Self-efficacy in using physiological signals of satiation.
* Correlation is significant at the 0.01 level.
