# Supplementary material for: Development and validation of the Multidimensional Internally Regulated Eating Scale (MIRES)
Source: PLoS One. 2020 Oct 8;15(10):e0239904. doi: 10.1371/journal.pone.0239904 (PMC7544044; doi:10.1371/journal.pone.0239904)
Supplement: S6 Table — (DOCX) [file pone.0239904.s008.docx]

# **S6 Table. Standardized regression coefficients (and R^2^) for the criterion validity of MIRES, IES-2, and ecSI-2.**

|  | MIRES^a^ | IES-2^a^ | ecSI-2^a^ | IT^b^ | FL^b^ | FE^b^ | SH^b^ | SS^b^ | SEH^b^ | SES^b^ | RI^b^ |
| --- | --- | --- | --- | --- | --- | --- | --- | --- | --- | --- | --- |
| BES | -0.42  (0.17)^c^ | -0.50  (0.25)^c^ | -0.18  (0.03)^c^ | -0.30  (0.09)^c^ | -0.31  (0.10)^c^ | -0.15  (0.02)^c^ | -0.33  (0.11)^c^ | -0.48  (0.23)^c^ | -0.34  (0.12)^c^ | -0.51  (0.26)^c^ | -0.36  (0.13)^c^ |
| RES | -0.17  (0.03)^c^ | -0.14  (0.02) ^c^ | -0.14  (0.02)^c^ | -0.10  (0.01)^c^ | -0.11  (0.01)^c^ | -0.13  (0.02)^c^ | -0.23  (0.05)^c^ | -0.16  (0.03)^c^ | -0.15  (0.02)^c^ | -0.14  (0.02)^c^ | -0.14  (0.02)^c^ |
| PCS | 0.41  (0.17)^c^ | 0.39  (0.15)^c^ | 0.48  (0.23)^c^ | 0.32  (0.10)^c^ | 0.27  (0.07)^c^ | 0.35  (0.12)^c^ | 0.39  (0.15)^c^ | 0.40  (0.16)^c^ | 0.39  (0.15)^c^ | 0.41  (0.17)^c^ | 0.39  (0.15)^c^ |
| SR | 0.26  (0.07)^c^ | 0.22  (0.05)^c^ | 0.17  (0.03)^c^ | 0.21  (0.04)^c^ | 0.17  (0.03)^c^ | 0.13  (0.02)^c^ | 0.15  (0.02)^c^ | 0.30  (0.09)^c^ | 0.20  (0.04)^c^ | 0.34  (0.12)^c^ | 0.25  (0.06)^c^ |
| SE | 0.26  (0.07)^c^ | 0.22  (0.05)^c^ | 0.23  (0.05)^c^ | 0.21  (0.05)^c^ | 0.18  (0.03)^c^ | 0.25  (0.06)^c^ | 0.18  (0.03)^c^ | 0.27  (0.07)^c^ | 0.25  (0.06)^c^ | 0.30  (0.10)^c^ | 0.25  (0.06)^c^ |
| BAS-2 | 0.50  (0.25)^c^ | 0.53  (0.28)^c^ | 0.59  (0.35)^c^ | 0.40  (0.16)^c^ | 0.48  (0.23)^c^ | 0.35  (0.12)^c^ | 0.40  (0.16)^c^ | 0.50  (0.25)^c^ | 0.45  (0.20)^c^ | 0.51  (0.26)^c^ | 0.51  (0.26)^c^ |
| SWLS | 0.30  (0.09)^c^ | 0.29  (0.09)^c^ | 0.42  (0.18)^c^ | 0.24  (0.06)^c^ | 0.22  (0.05)^c^ | 0.24  (0.06)^c^ | 0.25  (0.06)^c^ | 0.29  (0.08)^c^ | 0.29  (0.08)^c^ | 0.30  (0.09)^c^ | 0.28  (0.08)^c^ |
| SISE | 0.34  (0.12)^d^ | 0.35  (0.12)^d^ | 0.40  (0.16)^d^ | 0.28  (0.08)^c^ | 0.34  (0.12)^c^ | 0.25  (0.06)^c^ | 0.28  (0.08)^c^ | 0.33  (0.11)^c^ | 0.31  (0.10)^c^ | 0.34  (0.11)^c^ | 0.33  (0.11)^c^ |
| BMI | -0.15  (0.02)^d^ | -0.21  (0.05)^d^ | -0.12  (0.01)^d^ | -0.18  (0.03)^c^ | -0.21  (0.05)^c^ | -0.04^e^  (<0.01)^c^ | -0.07  (<0.01)^c^ | -0.16  (0.02)^c^ | -0.13  (0.02)^c^ | -0.19  (0.04)^c^ | -0.17  (0.03)^c^ |
| MWC | -0.16  (0.03)^d^ | -0.21  (0.04)^d^ | -0.16  (0.03)^d^ | -0.15  (0.02)^c^ | -0.14  (0.02)^c^ | -0.07  (<0.01)^c^ | -0.14  (0.02)^c^ | -0.14  (0.02)^c^ | -0.16  (0.02)^c^ | -0.17  (0.03)^c^ | -0.17  (0.03)^c^ |
| WCS (N=504) | -0.22  (0.05)^d^ | -0.27  (0.07)^d^ | -0.09  (<0.01)^d^ | -0.21  (0.04)^c^ | -0.23  (0.06)^c^ | -0.08^e^  (<0.01)^c^ | -0.19  (0.04)^c^ | -0.25  (0.06)^c^ | -0.17  (0.03)^c^ | -0.25  (0.06)^c^ | -0.22  (0.05)^c^ |

MIRES: Multidimensional Internally Regulated Eating Scale, IES-2: Intuitive Eating Scale-2, ecSI-2: Eating Competence Satter Inventory 2, IT: Internal trust, FL: Food legalizing, FE: Food enjoyment, SH: Sensitivity to physiological signals of hunger, SS: Sensitivity to physiological signals of satiation, SEH: Self-efficacy in using physiological signals of hunger, SES: Self-efficacy in using physiological signals of satiation, RI: Reflective items, BES: Binge Eating Scale, RES: Restrictive Eating Scale, PCS: Proactive Coping Scale, SR: Satiety Responsiveness, SE: Slowness in Eating, BAS-2: Body Appreciation Scale-2, SWLS: Satisfaction With Life Scale, SISE: Single Item Self-Esteem Scale, BMI: Body Mass Index, MWC: Maximal Weight Change, WCS: Weight Cycling Severity.
^a^ Summed score of all items included in the scale.
^b^ Latent factor as measured by observed items taking into account measurement error.
^c^ Values obtained with SEM.
^d^ Values obtained with linear regression.

^e^ p > 0.05

Controlling for variables such as gender, age, BMI, and currently following eating rules had minor impact on the results, thus, non-corrected values are displayed.
