# Supplementary material for: Development and validation of the Multidimensional Internally Regulated Eating Scale (MIRES)
Source: PLoS One. 2020 Oct 8;15(10):e0239904. doi: 10.1371/journal.pone.0239904 (PMC7544044; doi:10.1371/journal.pone.0239904)
Supplement: S7 Table — (DOCX) [file pone.0239904.s009.docx]

# **S7 Table. Standardized regression coefficients (and R^2^) for the criterion validity of SH, SS, SEH, SES (full subscales including neutral, emotional, external contexts) *vs*. the neutral counterpart of each subscale.**

| Outcome | SH^b^ | SH:  Neutral^b^ | SS^b^ | SS:  Neutral^b^ | SEH^b^ | SEH:  Neutral^b^ | SES^b^ | SES:  Neutral^b^ | MIRES^a^  (45 items) | MIRES^a^  (21 items) |
| --- | --- | --- | --- | --- | --- | --- | --- | --- | --- | --- |
| BES | -00.33  (0.11)^c^ | -00.29  (0.08)^c^ | -0.48  (0.23)^c^ | -0.44  (0.19)^c^ | -0.34  (0.12)^c^ | -0.31  (0.10)^c^ | -0.51  (0.26)^c^ | -0.45  (0.21)^c^ | -0.42  (0.17)^c^ | -0.39  (0.15)^c^ |
| RES | -0.23  (0.05)^c^ | -0.24  (0.06)^c^ | -0.16  (0.03)^c^ | -0.19  (0.04)^c^ | -0.15  (0.02)^c^ | -0.18  (0.03)^c^ | -0.14  (0.02)^c^ | -0.16  (0.03)^c^ | -0.17  (0.03)^c^ | -0.19  (0.04)^c^ |
| PCS | 0.39  (0.15)^c^ | 0.33  (0.11)^c^ | 0.40  (0.16)^c^ | 0.40  (0.16)^c^ | 0.39  (0.15)^c^ | 0.34  (0.12)^c^ | 0.41  (0.17)^c^ | 0.39  (0.16)^c^ | 0.41  (0.17)^c^ | 0.42  (0.17)^c^ |
| SR | 0.15  (0.02)^c^ | 0.12  (0.01)^c^ | 0.30  (0.09)^c^ | 0.25  (0.06)^c^ | 0.20  (0.04)^c^ | 0.17  (0.03)^c^ | 0.34  (0.12)^c^ | 0.27  (0.07)^c^ | 0.26  (0.07)^c^ | 0.23  (0.05)^c^ |
| SE | 0.18  (0.03)^c^ | 0.12  (0.01)^c^ | 0.27  (0.07)^c^ | 0.21  (0.04)^c^ | 0.25  (0.06)^c^ | 0.19  (0.04)^c^ | 0.30  (0.10)^c^ | 0.24  (0.06)^c^ | 0.26  (0.07)^c^ | 0.24  (0.06)^c^ |
| BAS-2 | 0.40  (0.16)^c^ | 0.32  (0.10)^c^ | 0.50  (0.25)^c^ | 0.43  (0.18)^c^ | 0.45  (0.20)^c^ | 0.38  (0.15)^c^ | 0.51  (0.26)^c^ | 0.45  (0.20)^c^ | 0.50  (0.25)^c^ | 0.49  (0.24)^c^ |
| SWLS | 0.25  (0.06)^c^ | 0.21  (0.04)^c^ | 0.29  (0.08)^c^ | 0.25  (0.06)^c^ | 0.29  (0.08)^c^ | 0.24  (0.06)^c^ | 0.30  (0.09)^c^ | 0.26  (0.07)^c^ | 0.30  (0.09)^c^ | 0.29  (0.08)^c^ |
| SISE | 0.28  (0.08)^c^ | 0.22  (0.05)^c^ | 0.33  (0.11)^c^ | 0.28  (0.08)^c^ | 0.31  (0.10)^c^ | 0.25  (0.07)^c^ | 0.34  (0.11)^c^ | 0.29  (0.08)^c^ | 0.34  (0.12)^d^ | 0.33  (0.11)^d^ |
| BMI | -0.07  (0.005)^c^ | -0.06  (0.004)^c^ | -0.16  (0.02)^c^ | -0.13  (0.02)^c^ | -0.13  (0.02)^c^ | -0.10  (0.01)^c^ | -0.19  (0.04)^c^ | -0.15  (0.02)^c^ | -0.15  (0.02)^d^ | -0.16  (0.02)^d^ |
| MWC | -0.14  (0.02)^c^ | -0.07  (0.005)^c^ | -0.14  (0.02)^c^ | -0.14  (0.02)^c^ | -0.16  (0.02)^c^ | -0.15  (0.02)^c^ | -0.17  (0.03)^c^ | -0.15  (0.02)^c^ | -0.16  (0.03)^d^ | -0.16  (0.03)^d^ |
| WCS (N=504) | -0.19  (0.04)^c^ | -0.16  (0.03)^c^ | -0.25  (0.06)^c^ | -0.20  (0.04)^c^ | -0.17  (0.03)^c^ | -0.17  (0.03)^c^ | -0.25  (0.06)^c^ | -0.21  (0.04)^c^ | -0.22  (0.05)^d^ | -0.22  (0.05)^d^ |

SH: Sensitivity to physiological signals of hunger, SS: Sensitivity to physiological signals of satiation, SEH: Self-efficacy in using physiological signals of hunger, SES: Self-efficacy in using physiological signals of satiation, MIRES: Multidimensional Internally Regulated Eating Scale, BES: Binge Eating Scale, RES: Restrictive Eating Scale, PCS: Proactive Coping Scale, SR: Satiety Responsiveness, SE: Slowness in Eating, BAS-2: Body Appreciation Scale-2, SWLS: Satisfaction With Life Scale, SISE: Single Item Self-Esteem Scale, BMI: Body Mass Index, MWC: Maximal Weight Change, WCS: Weight Cycling Severity0.

^a^ Summed score of all items included in the scale.
^b^ Latent factor as measured by observed items taking into account measurement error.
^c^ Values obtained with SEM.
^d^ Values obtained with linear regression.
