# Supplementary material for: Development and validation of the Multidimensional Internally Regulated Eating Scale (MIRES)
Source: PLoS One. 2020 Oct 8;15(10):e0239904. doi: 10.1371/journal.pone.0239904 (PMC7544044; doi:10.1371/journal.pone.0239904)
Supplement: S9 Table — (DOCX) [file pone.0239904.s011.docx]

# **S9 Table. Bivariate correlations of summed scores of MIRES (21 items), RI, and MIRES subscales with IES-2 and ecSI-2.**

|  | MIRES | RI | IT | FL | FE | SH: Neutral | SS: Neutral | SEH: Neutral | SES: Neutral |
| --- | --- | --- | --- | --- | --- | --- | --- | --- | --- |
| IES-2 | 0.66^*^ | 0.66^*^ | 0.54^*^ | 0.55^*^ | 0.32^*^ | 0.48^*^ | 0.59^*^ | 0.53^*^ | 0.63^*^ |
| UPE | 0.23^*^ | 0.31^*^ | 0.20^*^ | 0.34^*^ | 0.03 | 0.19^*^ | 0.16^*^ | 0.19^*^ | 0.15^*^ |
| EPR | 0.42^*^ | 0.38^*^ | 0.35^*^ | 0.38^*^ | 0.16^*^ | 0.26^*^ | 0.39^*^ | 0.29^*^ | 0.43^*^ |
| RHSC | 0.68^*^ | 0.68^*^ | 0.56^*^ | 0.46^*^ | 0.38^*^ | 0.52^*^ | 0.60^*^ | 0.61^*^ | 0.64^*^ |
| BFCC | 0.41^*^ | 0.37^*^ | 0.29^*^ | 0.22^*^ | 0.35^*^ | 0.30^*^ | 0.38^*^ | 0.33^*^ | 0.41^*^ |
| ecSI-2 | 0.67^*^ | 0.65^*^ | 0.50^*^ | 0.48^*^ | 0.51^*^ | 0.55^*^ | 0.57^*^ | 0.57^*^ | 0.56^*^ |
| EatAtt | 0.70^*^ | 0.70^*^ | 0.50^*^ | 0.56^*^ | 0.45^*^ | 0.57^*^ | 0.59^*^ | 0.59^*^ | 0.58^*^ |
| FoodAccept | 0.39^*^ | 0.38^*^ | 0.28^*^ | 0.26^*^ | 0.39^*^ | 0.30^*^ | 0.31^*^ | 0.32^*^ | 0.31^*^ |
| IntReg | 0.59^*^ | 0.59^*^ | 0.47^*^ | 0.46^*^ | 0.36^*^ | 0.47^*^ | 0.50^*^ | 0.48^*^ | 0.50^*^ |
| ContSkills | 0.52^*^ | 0.46^*^ | 0.38^*^ | 0.29^*^ | 0.46^*^ | 0.45^*^ | 0.45^*^ | 0.45^*^ | 0.43^*^ |

MIRES: Multidimensional Internally Regulated Eating Scale, RI: Reflective items, IT: Internal trust, FL: Food legalizing, FE: Food enjoyment, SH: Sensitivity to physiological signals of hunger, SS: Sensitivity to physiological signals of satiation, SEH: Self-efficacy in using physiological signals of hunger, SES: Self-efficacy in using physiological signals of satiation, IES-2: Intuitive Eating Scale-2, UPE: Unconditional Permission to Eat, EPR: Eating for Physical Rather Than Emotional Reasons, RHSC: Reliance on Hunger and Satiety Cues, BFCC: Body Food Choice Congruence, ecSI-2: Eating Competence Satter Inventory 2, EatAtt: Eating Attitudes, FoodAccept: Food Acceptance, IntReg: Internal Regulation, ContSkills: Contextual Skills.
* Correlation is significant at the 0.01 level.
