# Supplementary material for: Development and validation of the Multidimensional Internally Regulated Eating Scale (MIRES)
Source: PLoS One. 2020 Oct 8;15(10):e0239904. doi: 10.1371/journal.pone.0239904 (PMC7544044; doi:10.1371/journal.pone.0239904)
Supplement: S10 Table — (DOCX) [file pone.0239904.s012.docx]

**S10 Table. Incremental variance in outcome measures accounted for by MIRES (21 items).**

|  | MIRES vs. IES-2 | | | | MIRES vs. ecSI-2 | | | |
| --- | --- | --- | --- | --- | --- | --- | --- | --- |
|  | R^2^  (IES-2) | R^2^ (IES-2 + MIRES) | ∆R^2^ | P value | R^2^  (ecSI-2) | R^2^ (ecSI-2 + MIRES) | ∆R^2^ | P value |
| BES^a^ | 0.249 | 0.256 | 0.007 | 0.002 | 0.032 | 0.166 | 0.134 | <0.001 |
| RES^a^ | 0.021 | 0.037 | 0.016 | <0.001 | 0.018 | 0.036 | 0.018 | <0.001 |
| PCS^a^ | 0.150 | 0.196 | 0.046 | <0.001 | 0.227 | 0.253 | 0.026 | <0.001 |
| SR^a^ | 0.047 | 0.060 | 0.013 | <0.001 | 0.028 | 0.066 | 0.038 | <0.001 |
| SE^a^ | 0.048 | 0.064 | 0.016 | <0.001 | 0.052 | 0.074 | 0.022 | <0.001 |
| BAS-2^a^ | 0.284 | 0.317 | 0.033 | <0.001 | 0.348 | 0.363 | 0.015 | <0.001 |
| SWLS^a^ | 0.085 | 0.104 | 0.019 | <0.001 | 0.178 | 0.178 | 0.000 | 0.815 |
| SISE^b^ | 0.119 | 0.138 | 0.019 | <0.001 | 0.157 | 0.165 | 0.008 | 0.001 |
| BMI^b^ | 0.045 | 0.046 | 0.001 | 0.516 | 0.014 | 0.024 | 0.010 | <0.001 |
| MWC^b^ | 0.043 | 0.044 | 0.001 | 0.207 | 0.025 | 0.031 | 0.006 | 0.006 |
| WCS^b^ (N=504) | 0.071 | 0.074 | 0.003 | 0.223 | 0.007 | 0.056 | 0.049 | <0.001 |

MIRES: Multidimensional Internally Regulated Eating Scale, IES-2: Intuitive Eating Scale-2, ecSI-2: Eating Competence Satter Inventory 2, BES: Binge Eating Scale, RES: Restrictive Eating Scale, PCS: Proactive Coping Scale, SR: Satiety Responsiveness, SE: Slowness in Eating, BAS-2: Body Appreciation Scale-2, SWLS: Satisfaction With Life Scale, SISE: Single Item Self-Esteem Scale, BMI: Body Mass Index, MWC: Maximal Weight Change, WCS: Weight Cycling Severity.
^a^ Values obtained with SEM.

^b^ Values obtained with hierarchical regression analysis.
