# Supplementary material for: Development and validation of the Multidimensional Internally Regulated Eating Scale (MIRES)
Source: PLoS One. 2020 Oct 8;15(10):e0239904. doi: 10.1371/journal.pone.0239904 (PMC7544044; doi:10.1371/journal.pone.0239904)
Supplement: S1 Appendix — (DOCX) [file pone.0239904.s013.docx]

# **S1 APPENDIX. The Multidimensional Internally Regulated Eating Scale (MIRES)**

**Permission and general guidelines**

The MIRES is freely available and no permission is required for its use. In case modifications are made to the scale, please specify them in detail and mention that these have been made by the users. We recommend using entire subscales instead of individual items to retain the psychometric properties of the subscales.

Users who want to conduct CFA or SEM using the complete formative MIRES model should also measure the six Reflective Items (RI) to warrant model identification.

To use the 21-item, simplified version of MIRES, assess items 10 to 21 with the neutral context introductory text only (see below in yellow highlight).

In the research outlined in this paper we administered the full 45-item MIRES in the following way:

- Internal trust, Food legalizing, and Food enjoyment were assessed first, before participants were asked to imagine themselves in any context to avoid spill-over effects from the contexts.
- The contexts were randomized and a new context was introduced only when all items from the previous one had been rated.
- Within each context, sensitivity items were assessed before the self-efficacy items because this is a more logical order given the temporal relationship between these constructs.
- Reflective items were assessed after the MIRES using the same general instructions and response scale.

**General instructions**

*Please indicate how true or untrue is each of the following statements for you.*

Internal trust

1. I am confident that my body can decide how much I eat
2. I am confident that my body can decide when I eat
3. I trust that my body can guide my eating

Food legalizing

1. I am relaxed about my relationship with food
2. I do not feel guilty if I occasionally overeat
3. I can eat all foods that I like without guilt

Food enjoyment

1. I like to savour my food by attending to its taste, smell, and texture
2. Paying attention at my food while eating gives me more satisfaction
3. I enjoy using all my senses to savour my food

Sensitivity to physiological signals of hunger*

1. I realize when my stomach is empty
2. I know when my body is telling me to eat
3. I recognise the hollow sensation in the stomach that signals hunger

Sensitivity to physiological signals of satiation*

1. I know when my body is telling me to stop eating
2. I can distinguish between appetite and hunger
3. I notice when my stomach is comfortably full

Self-efficacy in using physiological signals of hunger*

1. I find it easy to let my hunger determine when I eat
2. I find it easy to listen to my body when it tells me to eat
3. I find it easy to rely on my hunger to tell me when to eat

Self-efficacy in using physiological signals of satiation*

1. I find it easy to stop eating once I feel comfortably satiated
2. I find it easy to stop eating when my body tells me I had enough
3. I find it easy to rely on my satiation feelings to tell me when to stop eating

*** Context introductory texts for the sensitivity and self-efficacy subscales:**

Neutral context:
“*In order to respond to the following statements, imagine a situation where you are calm, relaxed, and without much distraction*”

Emotional context:
“*In order to respond to the following statements, imagine a situation where you are sad, lonely, or bored*”

External context:
“*In order to respond to the following statements, imagine a situation where you are distracted by something*”

**Reflective items (RI) of the MIRES**

1. I have a general tendency to eat in response to my internal hunger and satiety signals
2. In deciding about eating, I just follow what my body tells me
3. I don’t make much of an issue out of my eating
4. I have a carefree eating style
5. I have a positive and relaxed relationship with food
6. I savour my food without any sabotaging thoughts

**Suggested response format**

7-point Likert-type response scale: 1 = “Completely untrue for me”, 2 = “Moderately untrue for me”, 3 = “Slightly untrue for me”, 4 = “Neither true nor untrue for me”, 5 = “Slightly true for me”, 6 = “Moderately true for me”, 7 = “Completely true for me”.
