# Supplementary material for: Development and validation of the Multidimensional Internally Regulated Eating Scale (MIRES)
Source: PLoS One. 2020 Oct 8;15(10):e0239904. doi: 10.1371/journal.pone.0239904 (PMC7544044; doi:10.1371/journal.pone.0239904)
Supplement: S2 Appendix — (DOCX) [file pone.0239904.s014.docx]

# **S2 APPENDIX. Modifications of the MIRES item pool during the scale development and validation process**

[Item pool modifications after evaluation by nutrition researchers 2](#_Toc50106489)

[Item pool modifications after expert feedback 10](#_Toc50106490)

[Modifications in construct definitions and item pool at the end of the preliminary work 18](#_Toc50106491)

[Item pool modifications after temporal stability assessment 24](#_Toc50106492)

[Final item pool modifications to simplify the MIRES 27](#_Toc50106493)

# Item pool modifications after evaluation by nutrition researchers

103 original items

53 items adjusted

27 items dropped

26 items added

102 final items

| **Sub-scales and Definitions** | | **Items** | | |
| --- | --- | --- | --- | --- |
| **Internal trust** | |  | | |
| Internal trust is defined as the tendency to trust the body’s innate abilities in eating. This trust applies to two aspects of eating. First, there is the trust that the body is able to eat in order to survive (trusting that the body can decide for itself when to start eating in order to avoid the harsh feelings of hunger). Second, there is the trust that the body can stop eating tasty and tempting food in order to avoid the fullness-accompanied discomfort. Individuals with internal trust are expected to have confidence and trust on their innate abilities for self-regulation, and avoid resorting on externally-imposed rules to guide eating behaviour, such as a diet or somebody’s self-imposed eating rules. | | 1. I trust my intuition to start and stop eating 2. I trust my body to tell me when to eat 3. I rather trust my body than rules in a diet plan 4. I prefer to trust my body to choose what, when, and how much I eat than self-imposed eating rules 5. I am confident that my body can decide how much I eat 6. I trust my body to tell me how much to eat 7. I dislike to self-impose rules on my eating habits that make me ignore my hunger 8. I trust my body to guide me towards foods that satisfy my nutritional needs 9. I am confident that I can get the energy and nutrients my body needs even without following specific guidelines 10. I distrust my instincts in choosing when, what, and how much to eat (R) 11. I trust my body to tell me what to eat | | |
| **Sensitivity to physiological signals of hunger and satiation under neutral conditions** | | | |  |
| Sensitivity to physiological signals of hunger and satiation under neutral conditions is defined as the ability to sense/perceive the signals that are naturally generated by the body in response to hunger and satiation, in situations when external influences, intense emotions, and other distractions are not salient. Individuals with high sensitivity under neutral conditions are expected to able to sense hunger signals (a hollow sensation in the stomach, growling noises, fatigue, etc.), satiation signals (a feeling of stomach fullness, feeling content, stomach distension etc.), and cues that signal the consumption of specific foods, in situations when external or emotional influences are not salient. | 1. I realize when my stomach is empty 2. I notice when hunger makes me feel irritated 3. I realize when I move from feelings of content to the first sensations of hunger 4. I recognise the signals that my body sends me when it wants a specific food 5. I realize when my body tells me to eat a specific food 6. I notice when my stomach is comfortably full 7. I notice when I start feeling content by eating 8. I notice when I have eaten more than was needed to satisfy my hunger 9. I find it hard to tell the difference between comfortably satiated and full (R) 10. I know when my body is telling me to eat 11. I know when my body is telling me to stop eating 12. I am puzzled by my stomach sensations (R) | | |  |
| **Sensitivity to physiological signals of hunger and satiation under emotional prompts** | | | |  |
| Sensitivity to physiological signals of hunger and satiation under emotional prompts is defined as the ability to sense/perceive the signals that are naturally generated by the body in response to hunger and satiation, in situations when emotional cues such as negative emotions are salient. Individuals with high sensitivity under emotional prompts are expected to able to sense hunger signals (a hollow sensation in the stomach, growling noises, fatigue, etc.), satiation signals (a feeling of stomach fullness, feeling content, stomach distension etc.), and cues that signal the consumption of specific foods, even when they experience negative emotions. | 1. I recognise how full I am even when I feel sad, stressed, lonely, bored, or excited 2. Even when I am distracted by intense emotions, I notice when my stomach is empty 3. I am unable to sense the difference between physical and emotional hunger (R) 4. Intense feelings do not overrule my ability to perceive how hungry I am 5. When I am emotional, I am unable to sense my body’s needs for specific foods (R) 6. Even when I feel intense emotions, I recognise the signals that my body sends me when it wants a specific food 7. I notice when my stomach is comfortably satiated, even when I am sad, stressed, lonely, bored, or excited 8. I am unable to sense the difference between satisfied and full, when I experience intense emotions (R) 9. I can distinguish between appetite and hunger, even when I am emotional 10. Even when I experience negative emotions, I recognise the hollow sensation in the stomach that signals hunger 11. I recognise the feeling of hunger, even when I experience negative emotions 12. I recognise how hungry I am even when I feel sad, stressed, lonely, bored, or excited 13. When I am excited, I am unable to feel when I have had enough of food (R) | | |  |
| **Sensitivity to physiological signals of hunger and satiation under external prompts** | | |  |  |
| Sensitivity to physiological signals of hunger and satiation under external prompts is defined as the ability to sense/perceive the signals that are naturally generated by the body in response to hunger and satiation, in situations when external influences are salient. Examples of such influences are the availability or palatability of food, social influences, distractions, consumption norms etc. Individuals with high sensitivity under external prompts are expected to able to sense hunger signals (a hollow sensation in the stomach, growling noises, fatigue, etc.), satiation signals (a feeling of stomach fullness, feeling content, stomach distension etc.), and cues that signal the consumption of specific foods, even when environmental influences are salient. | 1. I am aware of my stomach sensations even when there is a lot going on around me 2. I notice how full I am, even when I eat with company 3. Even when I am in a busy environment, I realize when I switch from feeling satiated to feeling hungry 4. I sense my hunger irrespectively of mealtime schedules 5. I only realize I am hungry when it is my regular mealtime (R) 6. I recognise the signals that my body sends me when it wants a specific food, even when there is a lot of food around me 7. I realize when my body tells me to eat a specific food, even when I am distracted 8. No matter if I am surrounded by tasty food, I can tell whether I am physically hungry or not 9. Even when there is a lot of tasty food on the table, I can sense when I am comfortably satiated 10. I notice when I am comfortably satiated in social eating occasions 11. I cannot tell the difference between satisfied and full when I eat with others (R) 12. I only realize how full I am after I have finished my portion (R) 13. I find it difficult to tell I am full before I finish my portion (R) 14. I cannot feel satiated when eating with others (R) | | |  |
| **Responsiveness to physiological signals of hunger and satiation under neutral conditions** | | |  |  |
| Responsiveness to physiological signals of hunger and satiation under neutral conditions is defined as the ability to make use of the physiological signals of hunger and satiation that the body produces in order to regulate food intake, in situations when external influences, intense emotions, and other distractions are not salient. Individuals with high responsiveness under neutral conditions are expected to initiate eating when hunger is sensed, be able to select foods that their body craves for, and cease eating upon the initial feelings of satiation, in situations when external or emotional influences are not salient. | 1. I rely on my hunger to tell me when to eat 2. I tend to ignore my hunger until it gets stronger (R) 3. When I start feeling hungry, I prefer to respond to this sensation by eating something 4. I listen to my body when looking for what to eat 5. I listen to my body when it tells me to eat a specific food, either healthy or unhealthy 6. I rely on my satiation feelings to tell me when to stop eating 7. I tend to stop eating once I feel comfortably satiated 8. I take into account my fullness when eating 9. What, when, and how much I eat is determined by my body | | |  |
| **Responsiveness to physiological signals of hunger and satiation under emotional prompts** | | |  |  |
| Responsiveness to physiological signals of hunger and satiation under emotional prompts is defined as the ability to make use of the physiological signals of hunger and satiation that the body produces in order to regulate food intake, in situations when emotional cues such as negative emotions are salient. Individuals with high responsiveness under neutral conditions are expected to initiate eating when hunger is sensed, be able to select foods that their body craves for, and cease eating upon the initial feelings of satiation, even when they experience negative emotions. | 1. I listen to my body when it tells me to eat, even if I experience negative feelings 2. I rely on my hunger to tell me when to eat, even when I feel sad, stressed, lonely, bored, or excited 3. I listen to my body when it tells me to eat a specific food, even when I am in a negative mood 4. When I am emotional, I still rely on the signals that my body sends me in order to choose what to eat 5. I try to avoid the feeling of physical discomfort that follows big meals, even when I am sad, stressed, lonely, bored, or excited 6. I consider my fullness while eating, even when I feel intense emotions such as excitement 7. Even when I feel intense emotions, I follow my bodily signals to decide what to eat 8. I do not ignore my hunger, even when I feel excited (R) | | |  |
| **Responsiveness to physiological signals of hunger and satiation under external prompts** | | |  |  |
| Responsiveness to physiological signals of hunger and satiation under external prompts is defined as the ability to make use of the physiological signals of hunger and satiation that the body produces in order to regulate food intake, in situations when external influences are salient. Examples of such influences are the availability or palatability of food, social influences, distractions, consumption norms etc. Individuals with high responsiveness under neutral conditions are expected to initiate eating when hunger is sensed, be able to select foods that their body craves for, and cease eating upon the initial feelings of satiation, even when environmental influences are salient. | 1. I listen to my body when it tells me to eat, even when there is a lot going on around me 2. I rely on my hunger to tell me when to eat, even when I am surrounded by a lot of tasty food 3. I let myself respond to my stomach sensations telling me when to eat irrespectively of regular mealtimes 4. I like to wait to feel a hunger signal before I start eating, even when it is the regular mealtime 5. Even when there is a variety of food available, I listen to what my body needs at the time 6. I listen to my body when it tells me to eat a specific food, even when there is a lot of tasty food around me 7. I listen to my body when it tells me I am comfortably satiated, even when I eat with others 8. Even if my plate is not empty, I like to stop eating when my body tells me I had enough 9. I tend to avoid the physical discomfort that follows big meals, even when I am served big portions 10. When I am served a big portion of tasty food, I cannot consider my fullness anymore (R) | | |  |
| **Positive appreciation of one’s body** | | |  |  |
| Positive appreciation of one's body is defined as the acceptance of the body given its imperfections by acknowledging the value of its good qualities and by putting more emphasis on health and well-being over physical appearance. Individuals with high positive appreciation of their bodies are expected to replace or overrule negative thoughts about their appearance with positive and respectful statements about the body and its good qualities. | 1. I can live with a few extra kilos, as long as I feel comfortable in my body 2. A well-functioning body is more important to me than its appearance 3. I appreciate the positive aspects of my appearance 4. I accept my body as it is, even if it is not perfect 5. I have a tendency to focus on the negative rather than the positive aspects of my body (R) 6. In general I am happy with my body 7. I can say at least three positive things about my body 8. My health is more important to me than my physical appearance 9. My well-being is more important to me than my physical appearance 10. I have a positive attitude towards my body, including all its flaws and strengths 11. When I look in the mirror I mainly focus on the negative parts of my body (R) | | |  |
| **Positive appreciation of food and eating** | | |  |  |
| Positive appreciation of food and eating is defined as having a healthy and relaxed relationship with food (free of food pre-occupations), and being able to value the pleasure and satisfaction from each eating experience independently of the food, or the amount of food, that is consumed (healthy or indulgent). Individuals with high positive appreciation of food and eating are expected to have no taboo foods that they forbid themselves to consume, and treat the consumption of indulgent food as an overwhelming experience filled with satisfaction, rather than a regretful situation followed by guilt. | 1. I am relaxed about my relationship with food 2. I allow myself to eat all kinds of food that I like, either healthy or unhealthy 3. I have some taboo foods, which I try to avoid (R) 4. I acknowledge that eating food that I like is a pleasurable experience, even when I eat more than I should 5. I can eat all foods that I like without feelings of guilt 6. I feel guilty when I eat more than I should (R) 7. I feel guilty when I eat specific indulgent foods (R) 8. I can be obsessed with eating specific foods (R) 9. Eating my favourite snacks is followed by feelings of pleasure and satisfaction 10. I do not feel restricted in my food choice 11. I can eat my favourite snacks without regret 12. I think of eating as something positive 13. I am afraid I will gain weight if I eat unhealthy food (R) 14. I am afraid I will gain weight if eat too much (R) | | |  |

# Item pool modifications after expert feedback

Items that refer to self-selection of food, which we decided to exclude due to limited evidence in the literature.

Items that were misclassified by at least one expert in the sorting task.

New items that we added after the sorting task.

Headings added in the neutral and emotional contexts.

102 initial items

22 items adjusted

45 items dropped

39 items added

96 final items

| **Sub-scales and Definitions** | | **Items** | | |
| --- | --- | --- | --- | --- |
| **Internal trust** | |  | | |
| Internal trust is defined as the tendency to trust the body’s innate abilities in eating. This trust applies to two aspects of eating. First, there is the trust that the body is able to eat in order to survive (trusting that the body can decide for itself when to start eating in order to avoid the harsh feelings of hunger). Second, there is the trust that the body can stop eating tasty and tempting food in order to avoid the fullness-accompanied discomfort. Individuals with internal trust are expected to have confidence and trust on their innate abilities for self-regulation, and avoid resorting on externally-imposed rules to guide eating behaviour, such as a diet or somebody’s self-imposed eating rules. | | 1. I trust my intuition to start and stop eating 2. I trust my body to tell me when to eat 3. I rather trust my body than dietary rules 4. I prefer to trust my body to choose what, when, and how much I eat than self-imposed eating rules 5. I trust my body to tell me how much to eat 6. I dislike to self-impose rules on my eating habits that make me ignore my hunger 7. I trust my body to guide me towards foods that satisfy my nutritional needs 8. I am confident that I can get the energy my body needs even without following specific guidelines 9. I distrust my instincts in choosing when and how much to eat (R) 10. I trust my body to tell me what to eat 11. I am confident that my body can decide how much I eat | | |
| **Sensitivity to physiological signals of hunger and satiation under neutral conditions** | | | |  |
| Sensitivity to physiological signals of hunger and satiation under neutral conditions is defined as the ability to sense/perceive the signals that are naturally generated by the body in response to hunger and satiation, in situations when external influences, intense emotions, and other distractions are not salient. Individuals with high sensitivity under neutral conditions are expected to able to sense hunger signals (a hollow sensation in the stomach, growling noises, fatigue, etc.), satiation signals (a feeling of stomach fullness, feeling content, stomach distension etc.), and cues that signal the consumption of specific foods, in situations when external or emotional influences are not salient. | *In order to respond to the statements in the coming pages, imagine a normal everyday situation where you are calm, relaxed, and without much distraction*   1. I realize when my stomach is empty 2. I notice when hunger makes me feel irritated 3. I realize when I move from feeling content to the first sensations of hunger 4. I recognise the signals that my body sends me when it wants a specific food 5. I realize when my body tells me to eat a specific food 6. I notice when my stomach is comfortably full 7. I notice when I have eaten more than was needed to satisfy my hunger 8. I find it hard to tell the difference between comfortably satiated and full (R) 9. I know when my body is telling me to eat 10. I know when my body is telling me to stop eating 11. I am puzzled by my stomach sensations (R) 12. I notice how various foods differentially affect my energy level 13. I notice how my energy level is changed by eating 14. I recognise the hollow sensation in the stomach that signals hunger 15. I can distinguish between appetite and hunger | | |  |
| **Sensitivity to physiological signals of hunger and satiation under emotional prompts** | | | |  |
| Sensitivity to physiological signals of hunger and satiation under emotional prompts is defined as the ability to sense/perceive the signals that are naturally generated by the body in response to hunger and satiation, in situations when emotional cues such as negative emotions are salient. Individuals with high sensitivity under emotional prompts are expected to able to sense hunger signals (a hollow sensation in the stomach, growling noises, fatigue, etc.), satiation signals (a feeling of stomach fullness, feeling content, stomach distension etc.), and cues that signal the consumption of specific foods, even when they experience negative emotions. | *In order to respond to the following statements, please consider a situation in which you experience intense emotions such as stress, sadness, loneliness, boredom, or excitement*   1. Even when I am distracted by intense emotions, I notice when my stomach is empty 2. I am unable to sense the difference between physical and emotional hunger (R) 3. Intense feelings do not overrule my ability to perceive how hungry I am 4. When I am emotional, I am unable to sense my body’s needs for specific foods (R) 5. Even when I feel intense emotions, I recognise the signals that my body sends me when it wants a specific food 6. I notice when my stomach is comfortably satiated, even when I am sad, stressed, lonely, bored, or excited 7. I find it hard to tell the difference between comfortably satiated and full(R) 8. I recognise the hollow sensation in the stomach that signals hunger 9. When I am excited, I am unable to feel when I have had enough of food (R) 10. I notice how various foods differentially affect my energy level 11. I notice how my energy level is changed by eating 12. I notice when I have eaten more than was needed to satisfy my hunger 13. I realize when my stomach is empty 14. I know when my body is telling me to stop eating 15. I realize when I move from feeling content to the first sensations of hunger 16. I notice when my stomach is comfortably full 17. I know when my body is telling me to eat | | |  |
| **Sensitivity to physiological signals of hunger and satiation under external prompts** | | |  |  |
| Sensitivity to physiological signals of hunger and satiation under external prompts is defined as the ability to sense/perceive the signals that are naturally generated by the body in response to hunger and satiation, in situations when external influences are salient. Examples of such influences are the availability or palatability of food, social influences, distractions, consumption norms etc. Individuals with high sensitivity under external prompts are expected to able to sense hunger signals (a hollow sensation in the stomach, growling noises, fatigue, etc.), satiation signals (a feeling of stomach fullness, feeling content, stomach distension etc.), and cues that signal the consumption of specific foods, even when environmental influences are salient. | 1. Even when I am in a busy environment, I realize when I switch from feeling satiated to feeling hungry 2. I realize I am hungry only when it is my regular mealtime (R) 3. I recognise the signals that my body sends me when it wants a specific food, even when there is a lot of food around me 4. I realize when my body tells me to eat a specific food, even when I am distracted 5. No matter if I am surrounded by tasty food, I can tell whether I am physically hungry or not 6. Even when there is a lot of tasty food on the table, I notice when my stomach is comfortably full 7. I find it hard to tell the difference between satisfied and full when I eat with others (R) 8. I realize how full I am only after I have finished my portion (R) 9. I find it difficult to tell I am full before I finish my portion (R) 10. I notice how various foods differentially affect my energy level, even when there is a lot of food around me 11. I notice how my energy level is changed by eating, even when I am distracted 12. I realize when my stomach is empty, even when I am distracted 13. I know when my body is telling me to eat, even when I am in a busy environment 14. I recognise the hollow sensation in the stomach that signals hunger irrespectively of mealtime schedules 15. I find it hard to tell I am full before I finish my portion (R) | | |  |
| **Responsiveness to physiological signals of hunger and satiation under neutral conditions** | | |  |  |
| Responsiveness to physiological signals of hunger and satiation under neutral conditions is defined as the ability to make use of the physiological signals of hunger and satiation that the body produces in order to regulate food intake, in situations when external influences, intense emotions, and other distractions are not salient. Individuals with high responsiveness under neutral conditions are expected to initiate eating when hunger is sensed, be able to select foods that their body craves for, and cease eating upon the initial feelings of satiation, in situations when external or emotional influences are not salient. | *In order to respond to the statements in the coming pages, imagine a normal everyday situation where you are calm, relaxed, and without much distraction*   1. It is easy to rely on my hunger to tell me when to eat 2. I tend to ignore my hunger until it gets stronger (R) 3. When I start feeling hungry, I prefer to respond to this sensation by eating something 4. I listen to my body when looking for what to eat 5. I listen to my body when it tells me to eat a specific food, either healthy or unhealthy 6. It is easy to rely on my satiation feelings to tell me when to stop eating 7. It is easy to stop eating once I feel comfortably satiated 8. I find it hard to take into account my fullness while eating (R) 9. What, when, and how much I eat is determined by my body 10. I find it easy to let my hunger determine when I eat 11. I find it hard to ignore my hunger 12. I find it hard to ignore my fullness while eating 13. I try to avoid the feeling of physical discomfort that follows big meals 14. It is easy to stop eating when my body tells me I had enough 15. It is easy to listen to my body when it tells me I am comfortably satiated 16. It is easy to listen to my body when it tells me to eat | | |  |
| **Responsiveness to physiological signals of hunger and satiation under emotional prompts** | | |  |  |
| Responsiveness to physiological signals of hunger and satiation under emotional prompts is defined as the ability to make use of the physiological signals of hunger and satiation that the body produces in order to regulate food intake, in situations when emotional cues such as negative emotions are salient. Individuals with high responsiveness under neutral conditions are expected to initiate eating when hunger is sensed, be able to select foods that their body craves for, and cease eating upon the initial feelings of satiation, even when they experience negative emotions. | *In order to respond to the following statements, please consider a situation in which you experience intense emotions such as stress, sadness, loneliness, boredom, or excitement*   1. It is easy to listen to my body when it tells me to eat 2. It is easy to rely on my hunger to tell me when to eat 3. I listen to my body when it tells me to eat a specific food, even when I am in a negative mood 4. When I am emotional, I still rely on the signals that my body sends me in order to choose what to eat 5. I try to avoid the feeling of physical discomfort that follows big meals 6. I find it hard to take into account my fullness while eating (R) 7. Even when I feel intense emotions, I follow my bodily signals to decide what to eat 8. I find it hard to ignore my hunger 9. It is easy to rely on my satiation feelings to tell me when to stop eating 10. It is easy to stop eating once I feel comfortably satiated 11. I find it easy to let my hunger determine when I eat 12. I find it hard to ignore my fullness while eating 13. It is easy to listen to my body when it tells me I am comfortably satiated 14. It is easy to stop eating when my body tells me I had enough | | |  |
| **Responsiveness to physiological signals of hunger and satiation under external prompts** | | |  |  |
| Responsiveness to physiological signals of hunger and satiation under external prompts is defined as the ability to make use of the physiological signals of hunger and satiation that the body produces in order to regulate food intake, in situations when external influences are salient. Examples of such influences are the availability or palatability of food, social influences, distractions, consumption norms etc. Individuals with high responsiveness under neutral conditions are expected to initiate eating when hunger is sensed, be able to select foods that their body craves for, and cease eating upon the initial feelings of satiation, even when environmental influences are salient. | 1. I listen to my body when it tells me to eat, even when there is a lot going on around me 2. Even when I am surrounded by a lot of tasty food, it is easy to rely on my hunger to tell me when to eat 3. I let myself respond to my stomach sensations telling me when to eat irrespectively of regular mealtimes 4. I like to wait to feel a hunger signal before I start eating, even when it is the regular mealtime 5. Even when there is a variety of food available, I listen to what my body needs at the time 6. I listen to my body when it tells me to eat a specific food, even when there is a lot of tasty food around me 7. It is easy to stop eating once I feel comfortably satiated, even when I eat with others 8. Even if my plate is not empty, it is easy to stop eating when my body tells me I had enough 9. I try to avoid the physical discomfort that follows big meals, even when I am served big portions 10. When I am served a big portion of tasty food, I cannot consider my fullness anymore (R) 11. It is easy to rely on my satiation feelings to tell me when to stop eating, even when there is a lot going on around me 12. It is easy to stop eating once I feel comfortably satiated, even when there is a lot of tasty food around me 13. When I am distracted I find it hard to take into account my fullness while eating (R) 14. I try to let my hunger guide my eating irrespectively of mealtime schedules 15. I find it hard to ignore my fullness while eating, even if my plate is not empty yet 16. I find it hard to ignore my hunger, even when I am distracted with other activities 17. It is easy to listen to my body when it tells me to eat, even if it is not my regular mealtime | | |  |
| **Positive appreciation of one’s body** | | |  |  |
| Positive appreciation of one's body is defined as the acceptance of the body given its imperfections by acknowledging the value of its good qualities and by putting more emphasis on health and well-being over physical appearance. Individuals with high positive appreciation of their bodies are expected to replace or overrule negative thoughts about their appearance with positive and respectful statements about the body and its good qualities. | 1. I can live with a few extra kilos, as long as I feel comfortable in my body 2. A well-functioning body is more important to me than its appearance 3. I appreciate the positive aspects of my appearance 4. I accept my body as it is, even if it is not perfect 5. I have a tendency to focus on the negative rather than the positive aspects of my body (R) 6. In general I am happy with my body 7. My health is more important to me than my physical appearance 8. My well-being is more important to me than my physical appearance 9. I have a positive attitude towards my body, including all its flaws and strengths 10. When I look in the mirror I mainly focus on the negative parts of my body (R) | | |  |
| **Positive appreciation of food and eating** | | |  |  |
| Positive appreciation of food and eating is defined as having a healthy and relaxed relationship with food (free of food pre-occupations), and being able to value the pleasure and satisfaction from each eating experience independently of the food, or the amount of food, that is consumed (healthy or indulgent). Individuals with high positive appreciation of food and eating are expected to have no taboo foods that they forbid themselves to consume, and treat the consumption of indulgent food as an overwhelming experience filled with satisfaction, rather than a regretful situation followed by guilt. | 1. I am relaxed about my relationship with food 2. I have some taboo foods, which I try to avoid (R) 3. I acknowledge that eating food that I like is a pleasurable experience, even when I eat more than I should 4. I can eat all foods that I like without feelings of guilt 5. I feel guilty when I eat more than I should (R) 6. I can be obsessed with eating specific foods (R) 7. Eating my favourite snacks is followed by feelings of pleasure and satisfaction 8. I do not feel restricted in my food choice 9. I can eat my favourite snacks without regret 10. I think of eating as something positive 11. I am afraid I will gain weight if I eat unhealthy food (R) 12. I am afraid I will gain weight if eat too much (R) | | |  |

# Modifications in construct definitions and item pool at the end of the preliminary work

86 initial items

17 items adjusted

43 items dropped

9 items added

52 final items

| **Sub-scales and Definitions** | | **Items** | |
| --- | --- | --- | --- |
| **Internal trust** | |  | |
| Internal trust is defined as the tendency to trust the body can manage the regulation of eating itself without the need for external or cognitive control. | | 1. I trust my intuition to start and stop eating 2. I trust my body to tell me when to eat 3. I trust my body to tell me how much to eat 4. I am confident that my body can decide how much I eat 5. I am confident that my body can decide when I eat 6. I trust that my body can guide my eating 7. My body can decide for itself when and how much to eat | |
| **Sensitivity to physiological signals of hunger under neutral conditions** | | | |
| Sensitivity to physiological signals of hunger under neutral conditions is defined as the ability to sense/perceive and interpret the signals that the body generates in response to hunger, when one is calm, relaxed, and without much distraction. | *Imagine that you are calm, relaxed, and without much distraction*   1. I realize when my stomach is empty 2. I know when my body is telling me to eat 3. I recognise the hollow sensation in the stomach that signals hunger | | |
| **Sensitivity to physiological signals of satiation under neutral conditions** | | | |
| Sensitivity to physiological signals of satiation under neutral conditions is defined as the ability to sense/perceive and interpret the signals that the body generates in response to satiation, when one is calm, relaxed, and without much distraction. | *Imagine that you are calm, relaxed, and without much distraction*   1. I notice when my stomach is comfortably full 2. I know when my body is telling me to stop eating 3. I can distinguish between appetite and hunger | | |
| **Sensitivity to physiological signals of hunger under emotional prompts** | | | |
| Sensitivity to physiological signals of hunger under emotional prompts is defined as the ability to sense/perceive and interpret the signals that the body generates in response to hunger, when one is sad, lonely, or bored. | *Imagine that you are sad, lonely, or bored*   1. I recognise the hollow sensation in the stomach that signals hunger 2. I realize when my stomach is empty 3. I know when my body is telling me to eat | | |
| **Sensitivity to physiological signals of satiation under emotional prompts** | | | |
| Sensitivity to physiological signals of satiation under emotional prompts is defined as the ability to sense/perceive and interpret the signals that the body generates in response to satiation, when one is sad, lonely, or bored. | *Imagine that you are sad, lonely, or bored*   1. I know when my body is telling me to stop eating 2. I notice when my stomach is comfortably full 3. I can distinguish between appetite and hunger | | |
| **Sensitivity to physiological signals of hunger under external prompts** | | |  |
| Sensitivity to physiological signals of hunger under external prompts is defined as the ability to sense/perceive and interpret the signals that the body generates in response to hunger, when one is distracted by something. | *Imagine that you are distracted by something*   1. I realize when my stomach is empty 2. I know when my body is telling me to eat 3. I recognise the hollow sensation in the stomach that signals hunger | | |
| **Sensitivity to physiological signals of satiation under external prompts** | | |  |
| Sensitivity to physiological signals of satiation under external prompts is defined as the ability to sense/perceive and interpret the signals that the body generates in response to satiation, when one is distracted by something. | *Imagine that you are distracted by something*   1. I notice when my stomach is comfortably full 2. I know when my body is telling me to stop eating 3. I can distinguish between appetite and hunger | | |
| **Self-efficacy in using physiological signals of hunger under neutral conditions** | | | |
| Self-efficacy in using physiological signals of hunger under neutral conditions is defined as the perception of ease (or difficulty) in using physiological signals of hunger to decide when to eat, when one is calm, relaxed, and without much distraction. | *Imagine that you are calm, relaxed, and without much distraction*   1. I find it easy to rely on my hunger to tell me when to eat 2. I find it easy to let my hunger determine when I eat 3. I find it easy to listen to my body when it tells me to eat | | |
| **Self-efficacy in using physiological signals of satiation under neutral conditions** | | | |
| Self-efficacy in using physiological signals of satiation under neutral conditions is defined as the perception of ease (or difficulty) in using physiological signals of satiation to decide how much to eat, when one is calm, relaxed, and without much distraction. | *Imagine that you are calm, relaxed, and without much distraction*   1. I find it easy to stop eating when my body tells me I had enough 2. I find it easy to rely on my satiation feelings to tell me when to stop eating 3. I find it easy to stop eating once I feel comfortably satiated | | |
| **Self-efficacy in using physiological signals of hunger under emotional prompts** | | | |
| Self-efficacy in using physiological signals of hunger under emotional prompts is defined as the perception of ease (or difficulty) in using physiological signals of hunger to decide when to eat, when one is sad, lonely, or bored. | *Imagine that you are sad, lonely, or bored*   1. I find it easy to listen to my body when it tells me to eat 2. I find it easy to rely on my hunger to tell me when to eat 3. I find it easy to let my hunger determine when I eat | | |
| **Self-efficacy in using physiological signals of satiation under emotional prompts** | | | |
| Self-efficacy in using physiological signals of satiation under emotional prompts is defined as the perception of ease (or difficulty) in using physiological signals of satiation to decide how much to eat, when one is sad, lonely, or bored. | *Imagine that you are sad, lonely, or bored*   1. I find it easy to stop eating when my body tells me I had enough 2. I find it easy to rely on my satiation feelings to tell me when to stop eating 3. I find it easy to stop eating once I feel comfortably satiated | | |
| **Self-efficacy in using physiological signals of hunger under external prompts** | | | |
| Self-efficacy in using physiological signals of hunger under external prompts is defined as the perception of ease (or difficulty) in using physiological signals of hunger to decide when to eat, when one is distracted by something. | *Imagine that you are distracted by something*   1. I find it easy to rely on my hunger to tell me when to eat 2. I find it easy to listen to my body when it tells me to eat 3. I find it easy to let my hunger determine when I eat | | |
| **Self-efficacy in using physiological signals of satiation under external prompts** | | | |
| Self-efficacy in using physiological signals of satiation under external prompts is defined as the perception of ease (or difficulty) in using physiological signals of satiation to decide how much to eat, when one is distracted by something. | *Imagine that you are distracted by something*   1. I find it easy to stop eating when my body tells me I had enough 2. I find it easy to rely on my satiation feelings to tell me when to stop eating 3. I find it easy to stop eating once I feel comfortably satiated | | |
|  | | |  |
|  |  | | |
| **Food legalizing** | | |  |
| Food legalizing is defined as having a relaxed relationship with food and particularly a relaxed attitude towards indulgent food | 1. I am relaxed about my relationship with food 2. I can eat all foods that I like without guilt 3. I do not feel guilty if I occasionally overeat 4. I can eat my favourite foods without regret | | |
| **Food enjoyment** | | |  |
| Food enjoyment is defined as the tendency to derive pleasure from eating by appreciating the sensory qualities of the food that is consumed. | 1. I take pleasure by eating with all my senses 2. I like to savour my food by attending to its taste, smell, and texture 3. I enjoy paying attention at how my food looks, smells, and tastes 4. Paying attention at my food while eating gives me more satisfaction 5. I enjoy using all my senses to savour my food | | |

# Item pool modifications after temporal stability assessment

52 initial items

0 items adjusted

7 items dropped

0 items added

45 final items

| **Internal trust** |  | | |
| --- | --- | --- | --- |
| 1. I am confident that my body can decide how much I eat 2. I am confident that my body can decide when I eat 3. I trust that my body can guide my eating | | | |
| **Sensitivity to physiological signals of hunger under neutral conditions** | | |  |
| *Imagine that you are calm, relaxed, and without much distraction*   1. I realize when my stomach is empty 2. I know when my body is telling me to eat 3. I recognise the hollow sensation in the stomach that signals hunger | | |  |
| **Sensitivity to physiological signals of satiation under neutral conditions** | | | |
| *Imagine that you are calm, relaxed, and without much distraction*   1. I notice when my stomach is comfortably full 2. I know when my body is telling me to stop eating 3. I can distinguish between appetite and hunger | | | |
| **Sensitivity to physiological signals of hunger under emotional prompts** | | |  |
| *Imagine that you are sad, lonely, or bored*   1. I recognise the hollow sensation in the stomach that signals hunger 2. I realize when my stomach is empty 3. I know when my body is telling me to eat | | |  |
| **Sensitivity to physiological signals of satiation under emotional prompts** | | |  |
| *Imagine that you are sad, lonely, or bored*   1. I know when my body is telling me to stop eating 2. I notice when my stomach is comfortably full 3. I can distinguish between appetite and hunger | | |  |
| **Sensitivity to physiological signals of hunger under external prompts** | |  |  |
| *Imagine that you are distracted by something*   1. I realize when my stomach is empty 2. I know when my body is telling me to eat 3. I recognise the hollow sensation in the stomach that signals hunger | | |  |
| **Sensitivity to physiological signals of satiation under external prompts** | |  |  |
| *Imagine that you are distracted by something*   1. I notice when my stomach is comfortably full 2. I know when my body is telling me to stop eating 3. I can distinguish between appetite and hunger | | |  |
| **Self-efficacy in using physiological signals of hunger under neutral conditions** | | |  |
| *Imagine that you are calm, relaxed, and without much distraction*   1. I find it easy to rely on my hunger to tell me when to eat 2. I find it easy to let my hunger determine when I eat 3. I find it easy to listen to my body when it tells me to eat | | |  |
| **Self-efficacy in using physiological signals of satiation under neutral conditions** | | |  |
| *Imagine that you are calm, relaxed, and without much distraction*   1. I find it easy to stop eating when my body tells me I had enough 2. I find it easy to rely on my satiation feelings to tell me when to stop eating 3. I find it easy to stop eating once I feel comfortably satiated | | |  |
| **Self-efficacy in using physiological signals of hunger under emotional prompts** | | |  |
| *Imagine that you are sad, lonely, or bored*   1. I find it easy to listen to my body when it tells me to eat 2. I find it easy to rely on my hunger to tell me when to eat 3. I find it easy to let my hunger determine when I eat | | |  |
| **Self-efficacy in using physiological signals of satiation under emotional prompts** | | |  |
| *Imagine that you are sad, lonely, or bored*   1. I find it easy to stop eating when my body tells me I had enough 2. I find it easy to rely on my satiation feelings to tell me when to stop eating 3. I find it easy to stop eating once I feel comfortably satiated | | |  |
| **Self-efficacy in using physiological signals of hunger under external prompts** | | |  |
| *Imagine that you are distracted by something*   1. I find it easy to rely on my hunger to tell me when to eat 2. I find it easy to listen to my body when it tells me to eat 3. I find it easy to let my hunger determine when I eat | | |  |
| **Self-efficacy in using physiological signals of satiation under external prompts** | | |  |
| *Imagine that you are distracted by something*   1. I find it easy to stop eating when my body tells me I had enough 2. I find it easy to rely on my satiation feelings to tell me when to stop eating 3. I find it easy to stop eating once I feel comfortably satiated | | |  |
| **Food legalizing** | |  |  |
| 1. I am relaxed about my relationship with food 2. I can eat all foods that I like without guilt 3. I do not feel guilty if I occasionally overeat | | |  |
| **Food enjoyment** | |  |  |
| 1. I like to savour my food by attending to its taste, smell, and texture 2. Paying attention at my food while eating gives me more satisfaction 3. I enjoy using all my senses to savour my food | | |  |

# Final item pool modifications to simplify the MIRES

45 initial items

0 items adjusted

24 items dropped

0 items added

21 final items

| **Internal trust** |  | | |
| --- | --- | --- | --- |
| 1. I am confident that my body can decide how much I eat 2. I am confident that my body can decide when I eat 3. I trust that my body can guide my eating | | | |
| **Sensitivity to physiological signals of hunger under neutral conditions** | | |  |
| *Imagine that you are calm, relaxed, and without much distraction*   1. I realize when my stomach is empty 2. I know when my body is telling me to eat 3. I recognise the hollow sensation in the stomach that signals hunger | | |  |
| **Sensitivity to physiological signals of satiation under neutral conditions** | | | |
| *Imagine that you are calm, relaxed, and without much distraction*   1. I notice when my stomach is comfortably full 2. I know when my body is telling me to stop eating 3. I can distinguish between appetite and hunger | | | |
| **Self-efficacy in using physiological signals of hunger under neutral conditions** | | |  |
| *Imagine that you are calm, relaxed, and without much distraction*   1. I find it easy to rely on my hunger to tell me when to eat 2. I find it easy to let my hunger determine when I eat 3. I find it easy to listen to my body when it tells me to eat | | |  |
| **Self-efficacy in using physiological signals of satiation under neutral conditions** | | |  |
| *Imagine that you are calm, relaxed, and without much distraction*   1. I find it easy to stop eating when my body tells me I had enough 2. I find it easy to rely on my satiation feelings to tell me when to stop eating 3. I find it easy to stop eating once I feel comfortably satiated | | |  |
| **Food legalizing** | |  |  |
| 1. I am relaxed about my relationship with food 2. I can eat all foods that I like without guilt 3. I do not feel guilty if I occasionally overeat | | |  |
| **Food enjoyment** | |  |  |
| 1. I like to savour my food by attending to its taste, smell, and texture 2. Paying attention at my food while eating gives me more satisfaction 3. I enjoy using all my senses to savour my food | | |  |
