# Supplementary material for: Development and validation of the Multidimensional Internally Regulated Eating Scale (MIRES)
Source: PLoS One. 2020 Oct 8;15(10):e0239904. doi: 10.1371/journal.pone.0239904 (PMC7544044; doi:10.1371/journal.pone.0239904)
Supplement: Attachment — Submitted filename: Response to reviewers.docx [file pone.0239904.s015.docx]

Manuscript number: PONE-D-19-26656

**General response**

We would like to thank both the reviewers and the academic editor for their helpful comments on our paper. We greatly appreciate the time and effort they spent to review our paper. We have attempted to address all comments in the revised manuscript. In addition, we have embedded our responses within the revision letter text.

Journal Requirements:

When submitting your revision, we need you to address these additional requirements:

1. Please ensure that your manuscript meets PLOS ONE's style requirements, including those for file naming. The PLOS ONE style templates can be found at <http://www.plosone.org/attachments/PLOSOne_formatting_sample_main_body.pdf> and <http://www.plosone.org/attachments/PLOSOne_formatting_sample_title_authors_affiliations.pdf>

We have now adapted our title, affiliations, contributorship, tables (and values with decimals in text), and file names to meet PLOS ONE’s style requirements.

2. In your Data Availability statement, you have not specified where the minimal data set underlying the results described in your manuscript can be found. PLOS defines a study's minimal data set as the underlying data used to reach the conclusions drawn in the manuscript and any additional data required to replicate the reported study findings in their entirety. All PLOS journals require that the minimal data set be made fully available. For more information about our data policy, please see <http://journals.plos.org/plosone/s/data-availability>.

Upon re-submitting your revised manuscript, please upload your study’s minimal underlying data set as either Supporting Information files or to a stable, public repository and include the relevant URLs, DOIs, or accession numbers within your revised cover letter. For a list of acceptable repositories, please see <http://journals.plos.org/plosone/s/data-availability#loc-recommended-repositories>. Any potentially identifying patient information must be fully anonymized.

Important: If there are ethical or legal restrictions to sharing your data publicly, please explain these restrictions in detail. Please see our guidelines for more information on what we consider unacceptable restrictions to publicly sharing data: <http://journals.plos.org/plosone/s/data-availability#loc-unacceptable-data-access-restrictions>. Note that it is not acceptable for the authors to be the sole named individuals responsible for ensuring data access.

We will update your Data Availability statement to reflect the information you provide in your cover letter.

We have added the following information in the revised cover letter. The data of Studies 1-7 have been uploaded in the OSF Repository (Creation date: 06/03/2020, URL: <https://osf.io/w3guh/?view_only=406839237c9f4bc09ff154d065c17da3>). For the purpose of this review, we provide the editors and reviewers with the view-only link to the project. The data will become publicly available once the paper is accepted for publication.

3. Your ethics statement must appear in the Methods section of your manuscript. If your ethics statement is written in any section besides the Methods, please move it to the Methods section and delete it from any other section. Please also ensure that your ethics statement is included in your manuscript, as the ethics section of your online submission will not be published alongside your manuscript.

Our ethics statement can be found in the Methods section of the paper.

4.  Thank you for including your ethics statement:  "The studies were conducted according to the guidelines laid down in the Declaration of Helsinki and complied with the code of conduct of Wageningen University. Written consent was obtained for all survey participants.".

For studies reporting research involving human participants, PLOS ONE requires authors to confirm that this specific study was reviewed and approved by an institutional review board (ethics committee) before the study began. Please provide the specific name of the ethics committee/IRB that approved your study, or explain why you did not seek approval in this case.

Once you have amended this/these statement(s) in the Methods section of the manuscript, please add the same text to the “Ethics Statement” field of the submission form (via “Edit Submission”).

For additional information about PLOS ONE ethical requirements for human subjects research, please refer to <http://journals.plos.org/plosone/s/submission-guidelines#loc-human-subjects-research>.

We have uploaded the retrospective approval from the Social Science Ethics Committee of Wageningen University in the submission system and we have adapted our ethics statement in the manuscript (lines 174-178) as well as in the submission form.

Reviewers' comments:

Reviewer #1: The authors present the results of an effort to develop a new measure of internally regulated eating. They make a reasonable case for the need for such a measure given extant measures of associated constructs. They mine the literature for dimensions that conceptually define internally regulated eating and develop a self-report measure that maps closely onto a domain-space defined by those dimensions. Their work is among the best I have read in terms of scale development and initial validation. Item generation and selection are thoughtful, and the psychometric analyses are thorough and informative. All in all, it is, in my view, a strong manuscript.

We thank the reviewer for his nice words. We are happy that he/she acknowledges our hard work.

Here are three minor considerations:

1. It is not clear to my what is to be gained from the series of one-factor models evaluated as described on pp. 12-13. The problem with these analyses is that do not examine the critical question of whether items written for a particular dimension uniquely indicate the intended dimension. It leads to reporting loadings in Table 1 that appear to be from a single model but in fact are from many small models. All I needed to see was the test of the full model, reported around the middle of p. 13, and my preference would be to see the loadings from that model in a supplemental table.

We agree with the reviewer that this part is confusing and perhaps less interesting for the reader. Thus, we removed it from the text (see edits in lines 161-162, 282-301, 375-377) and we added the requested table in the Supplementary material (S1 Table).

2. I understand the context distinction, but building it into the measures significantly increases the length and complexity of the measure if used as the authors intend. The question is whether information about internally regulated eating from specific contexts offers predictive advantages in hypothesis tests. If, as an example, one looks at the dieters-non-dieters comparisons in Table S1, Cohen's ds are virtually identical for SS and SES and not substantially different for SH and SEH. In terms of the latter two, it is notable that the inconsistent d is for the emotional context, which seems to be operating somewhat differently than the other two here and in other results. Similarly, the stability coefficients in Table 4 are very similar within dimension across context (e.g., .57, .64 and .62 for SH). Scores are collapsed across context within dimension in Tables S4 and S5, making it impossible to determine whether predictive or incremental validity varies as a function of context. In short, I think the authors need to make a stronger case for building context into the measure. My recommendation would be to, if at all possible, drop the distinction.

We agree with the reviewer that the context distinction makes our scale lengthier and more difficult to use. Thus, it makes sense to examine whether the addition of three contexts has predictive advantages over the neutral counterparts of the SH, SS, SEH, and SES subscales. However, we believe that Cohen’s ds and stability coefficients are not appropriate indicators of the contexts’ predictive advantages. To test this empirically we conducted additional analyses with the data of Study 7. Specifically, we tested whether the full subscales (SH, SS, SEH, and SES), which included all three contexts each, account for a larger amount of variance in each outcome measure compared to the neutral counterpart of each subscale. We addressed this issue in lines 773-789 and we added supplementary tables (S7, S8, S9, S10) and a figure (S2 Fig) to present the relevant results. Since the 45-item and 21-item versions of the scale are comparable we leave it to the user to decide which version to use depending on their specific interests (see also edits in lines 848-854 and S1 Appendix).

3. One issue for measures like this is how they should be scored. For the validity analyses, the authors consider both a total score and a score by dimension (ignoring context). My inference is that they would favor either scoring depending on research question. It is worth noting that researchers sometimes use only a subset of subscales from a measure like this. When the first-order factors are reflective indicators of the general factor, a total score can be meaningfully interpreted. In the case of formative indicators, as the authors have cast the first-order factors, that is not the case. The general factor is not fully defined unless all of the formative indicators are included in a composite.

We thank the reviewer for raising this important issue about the use of our scale. We now clarify this in lines 840-848.

A very minor matter: On p. 33, just before the general discussion, the authors suggest differences that imply comparisons of correlation coefficients, which they did not do. "Outperformed" implies a significantly larger coefficient, which requires a test comparing them.

We changed the phrasing in lines 768-770.

One somewhat related and even more minor matter: Please include the actual coefficients for all pairwise rs in Table 5. The use of "NS" in a table like this is a meta-analyst's nightmare and, generally speaking, unnecessarily hides information that need not be hidden.

We replaced the “NS” with the actual values as requested. We also corrected the values in the third column (correlations of IES-2) because we realized that the ones we had included in the original version were from an adapted version of IES-2 (in which all reverse-scored items had been excluded).

These matters are very minor and do not detract from my overall positive view of the manuscript. I congratulate the authors on a fine effort.

Once again, we thank the reviewer for his particularly useful comments that have helped us to improve our paper and scale substantially.

Reviewer #2: The authors report on the development of a new measure (MIRES). My first impression is that this is an extremely long paper, for its content. As such, it loses focus and clarity about its aims. By the end, I was not clear what psychological or clinical issue the authors were addressing, or what the implications of the work would be.

We thank the reviewer for the time spent on reviewing our paper and for the detailed comments. In this paper we present a large number of studies and analyses that we conducted for the thorough development and validation of the MIRES. We believe that all the information we provide is necessary and useful for the users of the scale. This prevents us from reducing the length of the paper substantially. However, we have made several efforts (already in the original version of the paper) to help the readers keep up with paper and its aims. Specifically, we use the following structure. First, we introduce the aim of the paper early on, in lines 60-73. Second, under the Methods section we present the overview of studies with their specific aims (lines 154-171), accompanied by Figure 1 (line 180), which is a graphical summary of the whole paper. Third, the results of each of the main studies are summarized in the discussion section of each study, followed by an overall wrap up in lines 801-823 of the General discussion section. Finally, we made some edits in lines 792-800 to remind readers of the main construct that we examine in this research.

More importantly, the presentation failed to meet a number of basic psychometric requirements in developing such a measure.

We regret to see that the reviewer is not satisfied with the amount of evidence we present on the scale’s psychometric properties. We believe this has to do with the way Studies 1-4 had been presented in the initial version of the manuscript. As we discuss later on, we now provide additional information on this preliminary work.

MAJOR ISSUES

There is a core problem with the reporting of the initial samples used in studies 3-7. The ages of the first two samples are substantially different, making the findings hard to compare. Without age-based norms for the original measure, comparability of scores or of factor structure is not safe to assume.

Starting with college samples is a common practice in scale development given the low costs and convenience of collecting such data. While many scale developers often settle with such data, we chose to go one step forward and test the properties of our scale in large community samples to increase the external validity of our research. We do not think that comparison of findings between college and community samples is problematic because in our studies we are mainly interested in the covariance between items not the variance within items. While means and standard deviations may vary depending on the sample, the covariance between items is not expected to do so (or at least not substantially).
Furthermore, the reason why we do not provide age-based or gender-based norms is because if we were to do this, we would first have to test our scale for measurement (in)variance across age groups or sexes. Since this would increase the length of the paper even more, we decided to leave this out for now and maybe present these data in another publication.

Studies 1-4 are simply not reported in anything like adequate detail. Where are the initial factor analyses that I presume must have been conducted? What factors emerged, using what methods? Was there replication? Internal consistency? Test-retest reliability? Most importantly, how do the authors justify a sample in studies 3 and 4 that are simply too small to allow for reliable factor derivation (80 items require a sample of 800 participants PER STUDY to ensure reliable outcomes)? In short, the derivation of a pool of 49 items is just not safe. Nor do we have any reason to assume that the factors that are addressed in studies 5-7 are in any way related to what was found in Studies 1-4.

We agree with the reviewer that these studies had been presented very briefly in the original paper. To enrich this section (see edits in lines 204-232), we added information on the rationale of dropping items, the sample size justification, and the most important findings of these studies, which helped us to bring the model to its current form.
The main reason why we do not present any hard data on these studies is because this preliminary work took place during the initial steps of developing our conceptual model. In the course of these studies our model was adjusted several times since we came across empirical findings we had not initially expected. Thus, should we want to add statistics on these studies we would have to present a long course of model adaptations and this would make the paper unnecessarily lengthier and more difficult to read. It would be challenging to keep the paper in its current concise form with a clear conceptual model to begin with. After all, in studies 5-7, which are the large-scale studies we conducted, we tested all important psychometric properties that are commonly reported in scale development (or even more). Thus, we believe that readers and users have all evidence needed to judge the psychometric quality of the scale.

For Study 5, the construct validity element is not clear. What were the t-values per measure, and what was the P value adopted? Why not use binomial regression to determine the key MIRES variables? And why use one simple measure like this (dieting/not dieting), when understanding such a complex construct? An appropriate index would have been to determine whether the MIRES is a better predictor than other measures of the same constructs, to show that the MIRES is a more clinically useful measure.

We added the t values in Table S2 as requested (we also found some small inaccuracies in the Cohen’s d column and we corrected them). As we now more clearly explain in lines 364-368, in Study 5 we tried to get preliminary evidence on the scale’s construct validity. Our aim was not to make an accurate prediction of dieting behaviour, but to show that non-dieters score higher in all MIRES subscales than dieters. This supports the very nature of the constructs we investigate in a broad sense, since internally regulated eating is by nature a non-diet eating style. Also, it was not the aim of this study to test the scale for criterion or incremental validity, as is suggested by the reviewer. Evidence on those types of validity are presented in Study 7 (Table S6 and Table 6).

If Study 6 really did use the same analyses as Study 5, where is the report of the CFA? If one was conducted, then the sample in Study 6 appears to be underpowered, even by the authors' relatively lax standards from study 6.

In lines 401-415 we describe the exact procedure that we followed to analyse the data of Study 6. We now clarify that we conducted individual analyses per subscale. Because we ran and compared up to seven CFA models per subscale (19 subscales), we decided not to report these results in a table, but rather to describe them briefly in lines 416-425, accompanied by Table 4. Since analysis was conducted per subscale the sample size was adequate for testing all first-order factor models. For testing the stability of second order factors the sample was indeed a bit small (4:1 ratio). We addressed this issue in lines 391-394.

In Study 6, the lack of temporal stability severely undermines the test-retest reliability of the measure - correlations on their own are not adequate, if scores change over time, which they do on two of the top three factors.

The reviewer is right that the scores of two subscales (IT and FL) change over time despite the fact that stability coefficients are adequate. This means that there is variation over time across the whole studied population. The change in means across time is a stricter test of stability than correlations and is not always reported by scale developers. However, we deem this important and so we have reported these results, hoping that readers will appreciate it. We made some edits in lines 456-460 and 813-815 in order to address this issue more explicitly.

Why were 4 items dropped at the end of Study 6? As this was the most underpowered study of the two in this section, it seems unclear why one would make that change.

As we now more explicitly explain in lines 435-446, we decided to drop four items in order to reduce the length of the scale and to have the same number of items per subscale. The reason why we dropped those items after the end of Study 6 was because we wanted to test the stability of the MIRES subscales first. The deletion of items was not based on findings from Study 6 but on the meaning of items, because all subscales were initially found to be stable on the basis of stability coefficients, which were adequate. The change in means was an extra analysis that we conducted at a later stage when writing the paper. By that time all studies had been completed.

In Study 7, the authors perform yet another CFA. Why? And what criteria are used for concluding that their measure was better than the others? That was not apparent from Table 5. In short, have the authors really just reinvented the wheel here, using a relatively long, psychometrically weak measure to do a job that was already done just as well by existing measures (in the case of Table 6, it looks as if the IES-2 already does this job for the core weight variables).

As we explain in lines 470-473, in Study 7 we test for the first time the full formative model of MIRES, including the six reflective items.
The criteria we used to decide whether our scale is better than the others included 1. the ability to predict outcome measures – Table S6 and Table 5 (descriptive comparison of scales) and 2. the incremental variance accounted for by MIRES – Table 6 (statistical testing). As can be clearly seen in Table 6, our scale accounts for significant amounts of extra variance in most outcome measures above and beyond the variance accounted for by IES-2 and ecSI-2 (see also edits in lines 715-720, which are intended to make this point clearer). Exceptions are the weight outcomes for which all three measures are relatively weak predictors.

The norms in Table 7 are meaningless if there is no validation.

Perhaps the reviewer has a different interpretation of norm scores than we do. We do not use norm scores in relation to predictive validity, i.e., expecting that individuals from different norm score categories would behave differently or show different outcomes, which would indeed require further validation. We use them from a descriptive point of view, to allow researchers to use them as reference and to compare between different groups or populations. Also, a subject’s raw score has little meaning, unless we know the subject’s position relative to others in some group/population. Thus, our norm scores provide the basis for comparing different groups or an individual with a group. We now clarify this in lines 742-745. As we explained earlier, we do not provide age-based or gender-based norms because this would require additional analyses that could perhaps be included in another publication. Finally, we re-calculated the norms based only on the data from the US sample because it is more useful to have country-based norms (see edits in lines 736-738 and Table 7).
