# Supplementary material for: Development and validation of the Multidimensional Internally Regulated Eating Scale (MIRES)
Source: PLoS One. 2020 Oct 8;15(10):e0239904. doi: 10.1371/journal.pone.0239904 (PMC7544044; doi:10.1371/journal.pone.0239904)
Supplement: Attachment — Submitted filename: Response to reviewers.docx [file pone.0239904.s016.docx]

Manuscript number: PONE-D-19-26656R1

**General response**
We want to thank the reviewers for taking the time to review our paper for a second time. We are happy that one of the reviewers was satisfied with the first revision and gave consent for acceptance of our paper. We equally appreciate the effort of the second reviewer to provide us with additional insights for improving our paper. We found some comments challenging, but we feel we have managed to address them all. The paper has been substantially revised in response to the reviewer comments. Most important changes are: (i) providing the details on studies 1-4, which we originally addressed more as pre-studies, but have now become an integral part of the “full history” of the development of the 21-item MIRES scale, (ii) strong recognition of the short version of the scale (21 items instead of 45 items), and (iii) shortening of the paper (from 12375 words to 11953 words) despite adding the requested material on studies 1-4, primarily obtained in the background information to the scale. We are grateful for the reviewer’s critical look at the paper and feel that the paper has benefited greatly from the reviewer’s comments. Please find our specific responses embedded in the text below.

Reviewers' comments:

Reviewer #2:

1. I do not understand the authors' conclusion that, having spent all that effort to demonstrate that the short and long version of the paper are equivalent, it should be up to the reader to decide which version to use. They present no rationale for having two versions, and I would recommend that they should focus on the shorter version, as that will eventually take a lot less time on the part of patients, clinicians and researchers.

In hindsight, we agree with the reviewer that most potential users may prefer to use the short version of the scale and that they should be able to access it quickly. In the revised manuscript, we now appreciate this from the beginning by including the following changes:

- We now mention the short version of the scale in the abstract (lines 30-33) and introduction (lines 92-95) so that readers expect this as the final outcome.
- We have made edits in lines 1031-1035 to contrast the two versions and show the advantage of the short version more explicitly.
- We moved Fig S2, which illustrates the simplified version, into the paper (see Fig 2 in lines 1037-1040) and moved Fig 2, which illustrates the full version, to the supplementary material (see S2 Fig). In the new Fig 2, we replaced the item codes with the actual items so that readers can see directly how the scale looks. S1 Appendix still provides information on how the full version was administered in our studies (to be used as a reference to the version we used in our studies) (lines 525-538). Although we are now directing users to the short version, we believe it is important to present the full version for transparency purposes.
- We explicitly advise potential users to go for the short version of the scale (lines 1107-1109).
- We skipped lines 1125-1130.

2. The authors state that they clarity their preferred scoring in lines 840-848, but they do not.

The main point raised in the first review was that users should not measure only a subset of subscales if they want to make conclusions about IRE style because all subscales of a formative construct need to be measured for the formative construct to be measured. This is the issue we addressed in lines 840-848 of the first revision. In the revised manuscript, we now explicitly refer to the appropriate scoring depending on the case (lines 1114-1119).

3. The paper remains excessively long, and the authors do not justify that length in their response letter. I did not find that the very minor changes alluded to made it any clearer what the aims, clinical issue or implications were.

We agree with the reviewer that the paper is long. We made additional efforts to shorten the paper. As we mentioned in the first revision, we believe all information about the scale’s properties is necessary and useful for the scale’s users; therefore, we tried to cut down parts of the paper that are less critical, i.e., information that can be traced back to existing literature. Effectively, we managed to reduce the length by 422 words and achieved that through the following changes:

- We removed examples of items and evidence on validity for the various measures we used in Study 7 to validate our scale (lines 754-881).
- We shortened the conceptual part by skipping background information and evidence of adaptivity for the IRE characteristics (lines 134-180), because a detailed overview of the conceptual model can be found in our theoretical paper that is freely accessible online. We now present only the definitions of the IRE characteristics and we explain how we operationalize them. In lines 126-128 we refer readers to the complete overview of the model.
- We skipped the description of the temporal stability assessment process because readers can find the full description of the method in the paper of Steenkamp & Van Trijp (lines 659-668).
- We skipped the section on norms (lines 982-1001); but see also point 4 below.
- We also made smaller edits throughout the paper to aid brevity and clarity (e.g., see lines 57-92).
- We skipped the wrap up of findings in the general discussion (lines 1059-1081) and adapted the abstract to contain all this information.

4. The lack of norms is defended as potentially making the paper longer (which would be more than offset if the authors had actually shortened the paper in response to previous feedback). However, the authors ignore the key issue that was identified - the lack of comparability between samples.

Even though we are not entirely sure, we believe that the reviewer aims to communicate that the whole scale development process should be conducted with comparable samples and that should non-comparable samples be used, measurement invariance of the model would have to be tested to ensure that the model holds its properties across the different samples.

We agree that measurement invariance testing is important when new scales are developed and perhaps should be conducted not only for different age groups but also for gender groups or other clinically relevant groups such as people with obesity. We now address the issue of comparability between samples in lines 1092-1105 and we have conducted additional analyses to provide preliminary evidence on the measurement invariance of our model across the age groups of interest (18-34 and 35-65). We realized that the sample sizes in our subgroups (in both studies 5 and 7) are too small to conduct typical measurement invariance testing (at least 980 participants per group are required to test the model), thus, we tried to find an alternative solution. Instead of testing for configural, metric, scalar, and residual invariance in a stepwise process as is usually done, we tried to test our model for full invariance at once by fixing all parameters to the values obtained from the full sample of study 7 and then fitting this model in each of the subgroups (18-34 and 35-65). In this way, the model had less parameters to be estimated and the sample sizes of our subgroups were adequate to provide reliable estimates. To warrant model identification we left only seven parameters free, the regression coefficients of the seven formative indicators. The fit of the model was good in both age groups providing preliminary evidence for the measurement invariance of our model. We further discuss this issue in the directions for future research (lines 1150-1153), where we also make a link to the development of norms.

5. The authors decline to provide key findings in Studies 1-4. The same applies later to other studies. That means that they are not replicable. That is a fundamental error in scientific communication, and I could not support publication of any such work, including this paper.

Per the reviewer’s request we have now included extensive information on these early studies as part of the “full history” of the MIRES development, both conceptually and in interaction with measurement. Studies 1-4 were initially considered pilot studies leading to the 52 initial items of the MIRES scale. These studies have helped us to develop that initial item set based on conceptual and empirical considerations. We absolutely agree with the reviewer on the importance of transparency and reproducibility of research. In the previous version we had focussed on the later stages and outcome of MIRES, and we now shift to describe the full history of the process with a stronger focus also on the early stages of scale development.

To allow readers to understand the full process of scale development that we followed, we added all details of Studies 1-4 in the paper and we made all data of this project freely accessible to the public (see edits in lines 30-37, 98-114, 206-215, 309-508). This will allow other researchers to replicate our work and to build further on it. Taking into account that there is always a degree of subjectivity in the scale development process (e.g., when selecting items to retain or to drop) we believe that readers are now fully equipped to understand the decisions we took during the scale’s trajectory by accessing the full data of the project.

6. The authors do not justify the lack of validation for their scores in Table 7, and they remain meaningless as a result.

The reviewer is right that descriptive kind of norms are less informative compared to validated norms that have been developed in relation to important clinical outcomes. After all, norms should develop from multiple studies and not from a single study. For these reasons and to help reduce the length of the paper we decided to skip the section on norms (lines 982-1001) and to discuss this issue in the discussion section as a promising direction for future research (lines 1152-1153).
