# Supplementary material for: Development and validation of the Multidimensional Internally Regulated Eating Scale (MIRES)
Source: PLoS One. 2020 Oct 8;15(10):e0239904. doi: 10.1371/journal.pone.0239904 (PMC7544044; doi:10.1371/journal.pone.0239904)
Supplement: Attachment — Submitted filename: Response to reviewers.docx [file pone.0239904.s017.docx]

**Response to reviewers**

Additional Editor Comments (if provided):

Dear Authors:

First of all, allow me to compliment you on your remarkable research, which is undoubtedly a contribution to the assessment of eating behaviors. I have reviewed your paper completely and the responses to the reviewers and I think you have done a good job. However, there is one comment on which I agree with reviewer 2 and that is the one referring to the length of the manuscript, although, more than the length, I have my misgivings about the structure.

In particular, I consider that all the studies are, in fact, part of one same study (with different samples and analyses), whose division into studies makes the manuscript somewhat complicated. I believe that there are parts of the report that are sufficient to be mentioned within the procedure (studies 1 to 4), putting the different pools of items as supplementary material, while, for the remaining studies, it would only be necessary to specify the different samples and the different analyses.

If you do not agree with modifying and unifying the structure of the study, please try to make it as integrated and simple as possible. I know that, at this stage of the work, it can be tedious to make this type of cosmetic transformation, however, it is necessary for me to make this recommendation since, in order for your work to have the scope it deserves, it is not only necessary to have the technical rigour (which your work already has), but it also needs to be friendly and easy to understand for the reader.

Response:

We would like to thank the editor for the time he spent on reading our original paper and the subsequent revisions and for his particularly kind and gratifying words regarding our work. We found the additional suggestions of the editor very useful and we believe the new edits we made have made the paper more “to the point” and easier to read. In line with the editor’s suggestion, we now present studies 1-7 as different parts of a single study with a unified Methods section and a unified Analysis and Results section. We refer to studies 1-4 as the preparatory work of this research (mentioned briefly under the Methods section) that led to the structure we used in large-scale testing of the MIRES. The data of all studies are still available online in the OSF repository (we changed the study numbers to descriptive titles so that readers can link more easily to our paper) and the versions of the MIRES items are now presented within a single document in the supplementary material (S2 Appendix). We also adapted Figure 1 so that it doesn’t refer specifically to studies and doesn’t include the sample sizes but is more descriptive of the properties of MIRES that we tested in this research. Finally, we have added a large paragraph in lines 948-985 of the Discussion section to summarize the main findings of this research because the individual discussions sections we used in the previous version have now been deleted. With these changes we managed to reduce the length of the paper by 2,619 words.

As an additional point, we now cite the latest version of our theoretical paper, which is also at the second round of revision in another journal (Palascha A, van Kleef E, de Vet E, van Trijp H. Internally Regulated Eating Style: A Comprehensive Theoretical Framework. OSF [Preprint, version 2]. 2019 [cited 2020 Sep 4]. Available from: https://osf.io/rmbft/ doi: 10.31219/osf.io/rmbft).

We hope these changes to have produced a paper that meets the criteria for publication at PlosOne, but we are happy to make more changes if the editor deems this necessary. We are looking forward to the editor’s response.
